# Synergistic Regulation by FoxO Signaling Pathway and Muscle Remodeling Defines the Adaptive Strategy of Largemouth Bass (*Micropterus salmoides*) Under Saline–Alkaline Stress

**DOI:** 10.3390/biology14091274

**Published:** 2025-09-16

**Authors:** Guoyang Liu, Di Peng, Biyuan Liu, Qiqun Cheng

**Affiliations:** 1 East China Sea Fisheries Research Institute, Chinese Academy of Fishery Sciences, Shanghai 200090, China; zhaozhaozz0602@outlook.com (G.L.); pengdi@ecsf.ac.cn (D.P.); deppliu@outlook.com (B.L.); 2 College of Fisheries and Life Science, Shanghai Ocean University, Shanghai 201306, China

**Keywords:** largemouth bass, saline–alkaline stress, growth performance, muscle, transcriptome, metabolomics

## Abstract

This study investigates the effects of saline, alkaline, and combined saline–alkaline water environments on the growth, muscle quality, gene expression, and metabolic profiles of largemouth bass (*Micropterus salmoides*). Juvenile fish were exposed to five water conditions for 60 days: freshwater (FW), saline water (SW, 10 ppt), alkaline water (AW, 15 mmol/L), and two saline–alkaline combinations (SAW-1: 4 ppt + 10 mmol/L; SAW-2: 6 ppt + 15 mmol/L). While growth rate was similar across groups, SAW-2 caused a significant decrease in survival rate and induced notable alterations in muscle texture and fiber structure. Transcriptomic analyses revealed group-specific enrichment of stress-responsive pathways. The FoxO signaling pathway acts as a central regulator of muscle maintenance and energy reallocation. The solute carrier gene *slc38a4* and *glula* (glutamine synthetase), both closely associated with ammonia detoxification via glutamine synthesis and transport, were upregulated under saline–alkaline stress, indicating enhanced capacity for nitrogen metabolism. In addition, two key regulators of muscle remodeling, *loc119898415* and *tbx18*, were significantly upregulated, suggesting a potential chromatin–transcription program underlying compensatory myogenesis and muscle fiber adaptation in response to environmental challenges. Metabolomic profiling showed an accumulation of osmoprotectants (betaine, taurine) in SW and SAW-2 groups, suggesting enhanced stress resistance. Multiomics integration further indicated coordinated regulation between lipid metabolism and insulin signaling, potentially mediated by the FoxO pathway. These results offer practical guidance for improving largemouth bass aquaculture under inland saline–alkaline conditions.

## Simple Summary

Saline–alkaline water environments pose a major threat to freshwater aquaculture by impairing fish growth, disrupting muscle structure, and interfering with ammonia excretion. Largemouth bass (*Micropterus salmoides*) is a widely farmed species in China, yet its tolerance to saline–alkaline stress and underlying physiological mechanisms remain unclear. In this study, we evaluated the growth, muscle texture, gene expression and metabolism of largemouth bass under different saline–alkaline water conditions. Our findings reveal that high carbonate alkalinity significantly impairs survival and muscle quality, while also activating key stress response pathways such as the FoxO signaling pathway. These results provide new insights into the adaptability of largemouth bass and offer a scientific basis for its application in saline–alkaline aquaculture.

## 1. Introduction

Saline–alkaline land refers to soil systems affected by salinity and alkalinity (e.g., NaCl, ${\mathrm{HCO}}_{3}^{-}$), and covers a significant portion of the global land area, especially in arid and semi-arid regions [1]. More than 800 million hectares of land worldwide are estimated to be affected by salinization and alkalization, with major distributions in Central Asia, Northern China, India, Australia, and parts of North America [2].

Saline–alkaline environments pose serious challenges to aquatic life. High salinity and alkalinity disrupt fish osmoregulation and ammonia excretion, leading to energy imbalance, growth retardation, and, in extreme cases, mortality [3]. In response to the harsh conditions of saline–alkaline water bodies, the strategy of *Fish farming to rehabilitate saline–alkaline land* has emerged as a promising ecological and livelihood-oriented solution. China has made remarkable progress in this domain, utilizing saline–alkaline tolerant fish species to both utilize and improve marginal land [4], contributing to local economies and ecological balance [5,6].

In recent years, researchers globally have investigated the mechanisms of saline–alkaline tolerance in dominant fish species found in saline–alkaline lakes, such as Magadi tilapia (*Oreochromis alacalicus*) [7], Qinghai Lake Naked Carp (*Gymnocypris przewalskii*) [8], and Amur ide (*Leuciscus waleckii*) [9], as well as in other fish species with strong tolerance to saline–alkaline conditions, including Gibel carp (*Carassius auratus gibelio*) [10], Nile tilapia (*O. niloticus*) [11], blackchin tilapia (*Sarotherodon melanotheron*) [12], and ‘California’ Mozambique tilapia (*O. mossambicus × O. urolepis hornorum*) [13]. However, with the development of the saline–alkaline aquaculture industry, currently farmed species cannot meet diverse regional demands, making it necessary to explore new high-quality aquaculture candidates. Most previous studies have examined either salinity or alkalinity stress independently, while the interactive effects of salinity and alkalinity on fish growth and adaptation remain poorly understood. The physiological and molecular responses of fish to combined saline–alkaline stress, particularly under carbonate-dominated conditions, warrant investigation.

The largemouth bass (*M. salmoides*. Perciformes: Percoidea: Centrarchidae: *Micropterus salmoides*), wildly farmed for aquaculture, possesses several advantages for aquaculture, including strong environmental adaptability, rapid growth, ease of handling, and a short farming cycle, but the full extent of its tolerance to saline–alkaline conditions and the underlying adaptive mechanisms remain unclear [14]. This study examines the effects of saline–alkaline water on largemouth bass in four key aspects: growth performance, muscle textural properties, gene expression, and metabolic profiles. The findings aim to provide a scientific basis for selecting suitable species for saline–alkaline aquaculture.

## 2. Materials and Methods

### 2.1. Fish Collection and Daily Management

The juvenile largemouth bass used in this experiment were selected from the ‘Excellent Bass No.1’ (You Lu No.1. Provided by Xinshun aquaculture technology Co., Ltd., Lianyungang, Jiangsu, China, Variety Approval No. GS01-004-2010), with an average body length of 72.6 ± 10.8 mm and a body weight of 8.85 ± 1.40 g. Approximately 1000 individuals were used in total. The fish were temporarily reared in two 500 L polyethylene tanks at a stocking density of 600 individuals per cubic meter. Culture water was sourced from underground municipal tap water, and was aerated for 24 h before use. The water temperature was maintained at 23.0 ± 0.4 °C, with a dissolved oxygen level of 6.0 ± 0.5 mg/L. During the temporary rearing period, the fish were fed once, with a feeding amount equivalent to 2% of their total body weight. Temporary care lasts for three days. This acclimation period was deemed sufficient because the fish had been maintained under similar freshwater conditions at the breeding facility, and no signs of stress or abnormal behavior were observed.

### 2.2. Acute Exposure to Salinity and Alkalinity

The salinity gradient was set at five levels: 8, 10, 12, 14, and 16 ppt. The alkalinity gradient was set at five levels: 25, 30, 35, 40 and 45 mmol/L, with a freshwater group serving as the control. The concentrations of salinity and carbonate alkalinity were expressed as parts per thousand (ppt) and mmol/L, respectively. Each group had three replicates, with 20 fish per replicate.

The preliminary saline–alkaline stress tolerance test lasted for 96 h. The experimental saline water was prepared using artificial sea salt (Changzhou Nolai Biotechnology Co., Ltd., Changzhou, Jiangsu, China), while the alkaline water was prepared using ${\mathrm{NaHCO}}_{3}$ (grade analytical reagent, Sinopharm Chemical Reagent Co., Ltd., Shanghai, China). All experimental water was aerated for 24 h before use and the water temperature was maintained at 23.2 ± 1.2 °C, with a dissolved oxygen level of 6.0 ± 0.5 mg/L. The average pH levels for each alkaline treatment were as follows: CA (carbonate alkalinity) 25 (8.90), CA30 (8.97), CA35 (9.04), CA40 (9.11), and CA45 (9.13). No buffering agents were added to actively control pH. The pH, temperature, and dissolved oxygen level were measured once daily and remained stable across replicates.

No feeding was provided during the acute exposure trials. Water temperature, dissolved oxygen, and pH levels were measured daily. Largemouth bass were considered dead when they lost equilibrium, ceased opercular movement, and showed no response to gentle tactile stimulation. Survival checks were conducted every 6 h at 06:00, 12:00, 18:00, and 24:00. Mortality in largemouth bass was recorded every 24 h and dead fish were promptly removed. Observers were aware of the group allocation during monitoring.

### 2.3. Long-Term Salinity and Alkalinity Stress Test

Based on the results of the acute toxicity experiment and the typical salinity and alkalinity levels in saline–alkaline areas, five experimental groups were established, with the freshwater group (FW, salinity 0.6ppt, alkalinity 1.8 ± 0.2 mmol/L) serving as the control. The experimental groups included a salinity group with a salinity level of 10 ppt (SW), an alkalinity group with a carbonate alkalinity of 15 mmol/L (AW), a low salinity–alkalinity interaction group with a salinity of 4 ppt and alkalinity of 10 mmol/L (SAW-1), and a high salinity–alkalinity interaction group with a salinity of 6 ppt and alkalinity of 15 mmol/L (SAW-2). These saline–alkaline levels were selected based on preliminary trials, which showed that higher combinations (e.g., 8 ppt + 15 mmol/L or 6 ppt + 20 mmol/L) caused severe mortality within 2–3 days. Thus, 4 + 10 and 6 + 15 represent the upper tolerable limits for juvenile largemouth bass under experimental conditions. A 60-day growth experiment was conducted across these five groups. Each group consisted of three replicates, with 15 largemouth bass per replicate (total 45 fish per treatment). All replicates were maintained in identical rearing systems, each consisting of a 125 L polyethylene tank with the same water source and aeration. The preparation of experimental water and rearing conditions were consistent with those described in Section 2.2.

During the experiment, fish were fed twice daily at 10:00 and 18:00, with each feeding amounting to 2% of the total body weight. An electric water changer was used to remove uneaten feed and feces from the tank bottom daily. To maintain water quality, 30% of the tank water was replaced every three days. Water temperature, pH, dissolved oxygen, and salinity were measured daily. The water temperature was maintained between 23.4 ± 1.2 °C, and pH values remained relatively stable across replicates. The average pH levels for each treatment were as follows: FW (7.82), SW (7.87), AW (8.70), SAW-1 (8.43), and SAW-2 (8.53). Survival checks were conducted every 6 h at 06:00, 12:00, 18:00, and 24:00 and dead fish were promptly removed. Mortality was recorded cumulatively across the entire experimental period. Observers were aware of the group allocation during monitoring.

To maintain a stable level of salinity and carbonate alkalinity throughout the experiment, the salinity of each saline treatment group and the alkalinity of each alkaline treatment group were monitored every three days using a salinometer (AS8012, SMART SENSOR, Dongguan, Guangdong, China, accuracy 0.1) and a titration-based carbonate alkalinity test kit (Zhejiang Lohand Enviroment Technology Co., Ltd., Hangzhou, Zhejiang, China). When the measured value deviated from the preset concentration by more than ±0.2 ppt or ±1 mmol/L, artificial sea salt or analytical-grade ${\mathrm{NaHCO}}_{3}$ were separately added to restore the target level. During routine water exchange (30% every three days), fresh water was pre-adjusted with sea salt or ${\mathrm{NaHCO}}_{3}$ to match the target salinity or alkalinity before being added to the tanks. This approach ensured minimal fluctuation in salinity and alkalinity across the 60-day experimental period.

### 2.4. Sample Collection

At day 60 of the experiment, 10 largemouth bass were randomly selected from each group. The fish were anesthetized on ice using the fish anesthetic MS-222 (tricaine methanesulfonate; Changsha Shanghe Biotechnology Co., Ltd., Changsha, Hunan, China.) at a concentration of 150 mg/L for deep anesthesia prior, and a 1 cm × 1 cm × 0.5 cm muscle sample was excised from the dorsal region using a surgical scalpel. After removing the skin, the samples were processed differently according to the specific experimental requirements:

(a) Placed into 2 mL centrifuge tubes and promptly transferred to a 4 °C refrigerator for storage (used for muscle textural properties).
(b) Fixed in 30% formaldehyde solution for 12 h (used for histology).
(c) Placed into 2 mL centrifuge tubes and promptly transferred to a −80 °C freezer for storage (used for transcriptome and metabolome).

The visceral mass and liver were also separated using a surgical scalpel and weighed by an analytical balance (Adventurer™ Semi–Micro, OHAUS International Trade Co., Ltd., Shanghai, China).

### 2.5. Growth Performance

At days 30 and 60 of the experiment, each fish was anesthetized using the fish anesthetic MS-222 at a concentration of 100 mg/L for mild anesthesia prior, followed by measurements of body length and weight. Calculations were performed for survival rate (SR), condition factor (CF), hepatosomatic index (HSI), and viscerasomatic index (VSI).

$$\begin{array}{cc} \mathrm{SR}(\%) & =\left(\frac{S}{N}\right)\times 100\%\\ \mathrm{HSI}(\%) & =\left(\frac{W_{h}}{W_{t}}\right)\times 100\%\\ \mathrm{VSI}(\%) & =\left(\frac{W_{v}}{W_{t}}\right)\times 100\% \end{array}$$

$$\begin{array}{cc} \mathrm{CF} & =\left(\frac{W_{t}}{L^{3}}\right)\times 100\% \end{array}$$

where *S* is the number of fish that survived up to sampling, *N* is the number of initial fish, $W_{t}$ is the final body weight (g) of the largemouth bass, *L* is the final body length (cm), $W_{h}$ is the final liver weight (g), and $W_{v}$ is the final viscera weight (g).

The median lethal concentration ($LC_{50}$) for saline–alkaline exposure was determined by Probit analysis. The number of deaths at each salinity–alkalinity concentration was used as the response frequency, and the exposure concentration (expressed as the logarithm, base 10) was taken as the independent variable. The dose–response data were fitted to a linear regression model after probit transformation of the observed mortality. The $LC_{50}$ value and its 95% confidence intervals were estimated from the concentration corresponding to a cumulative response probability of 0.5.

### 2.6. Muscle Textural Properties and Sections

#### 2.6.1. Texture Analyzer Conditions

After operation (Section 2.4 list (a)), the muscle samples were acclimated at room temperature (25 °C) for 30 min. Subsequently, their textural properties were measured using a TMS-Pro texture analyzer (Food Technology Corporation, Sterling, VA, USA). The testing parameters were set as follows: a return distance of 25 mm, a deformation of 50%, a test speed of 60 mm/min, and a trigger force of 1.5 N.

#### 2.6.2. Histology Preparation

After operation (Section 2.4 list (b)), the fixed tissues underwent dehydration through a graded ethanol series, followed by clearing with xylene to render them translucent. Tissues were infiltrated with molten paraffin wax at approximately 60 °C, embedded to form paraffin blocks, and sectioned into 4–8 µm thick slices using a microtome. The sections were mounted onto glass slides and dried in a 45 °C incubator.

For H&E (hematoxylin–eosin) staining, sections were deparaffinized in xylene, rehydrated through descending ethanol concentrations to water, stained with hematoxylin to visualize nuclei, differentiated in acid alcohol, neutralized in ammonia water, counterstained with eosin for cytoplasmic and extracellular matrix visualization, dehydrated through ascending ethanol concentrations, cleared in xylene, and finally mounted with coverslips using a suitable medium.

### 2.7. Analysis of Transcriptome

#### 2.7.1. RNA Extraction and Library Construction

After operation (Section 2.4 list (c)), total RNA was extracted from muscle tissues using TRIzol reagent (Invitrogen, Carlsbad, CA, USA), and the integrity and concentration were assessed with a NanoDrop spectrophotometer and Agilent 2100 Bioanalyzer (Santa Clara, CA, USA). RNA-seq libraries were constructed following Illumina’s standard protocol, including mRNA enrichment, fragmentation, cDNA synthesis, and PCR amplification. The libraries were sequenced on an Illumina platform to generate paired-end reads.

#### 2.7.2. Quality Control and Read Mapping

Raw reads were first assessed for quality using FastQC (v0.11.9). Adapter sequences and low-quality bases were then removed using Trimmomatic (v0.39), and the resulting high-quality clean reads were used for downstream analysis. Clean reads were mapped to the largemouth bass reference genome (Micropterus_salmoides.GCF_014851395.1) using HISAT2 (v2.0.4), and transcript assembly was performed using StringTie (v2.2.1).

#### 2.7.3. Identification of Differentially Expressed Genes (DEGs)

Differential gene expression analysis was conducted using the DESeq2 package (v1.30.1) in R. Genes were considered significantly differentially expressed if they met the following criteria:

Adjusted *p*-value (Benjamini–Hochberg-corrected) < 0.05, equivalent to *Q* < 0.05 or FDR < 0.05,

$$\left|\log_{2}\mathrm{FC}\right|>1$$

Gene expression levels were normalized as transcripts per million (TPM) as appropriate.

#### 2.7.4. GO Enrichment and KEGG Pathway Enrichment

GO enrichment analysis of DEGs was performed using the clusterProfiler package (v4.0.5) in R. Significantly enriched GO terms (biological process, cellular component, and molecular function) were identified based on *Q* < 0.05.

KEGG pathway enrichment was also conducted with clusterProfiler, mapping DEGs to the Kyoto Encyclopedia of Genes and Genomes (KEGG) database. Pathways with *Q* < 0.05 were considered significantly enriched.

#### 2.7.5. qRT-PCR Validation

To validate RNA-seq results, six differentially expressed genes were selected for qRT-PCR analysis using the QuantStudio™ 5 System (Thermo Fisher Scientific, Waltham, MA, USA) and HiScript® Q RT SuperMix (Vazyme, Bio-Rad, Hercules, CA, USA). Primers were designed with Primer 5 software and synthesized by Guangzhou Genedenovo Biotechnology Co., Ltd. (Guangzhou, Guangdong, China). Relative gene expression was calculated using the $2^{-\Delta \Delta Ct}$ method with *gapdh* as the reference gene. Primer sequences are listed in (Table 1).

### 2.8. Analysis of Metabolome

#### 2.8.1. Metabolomic Sample Preparation and Instrumental Analysis

After operation (Section 2.4 (c)), approximately 100 mg of muscle tissue was extracted with methanol/acetonitrile/water (2:2:1, *v*/*v*) and processed by ultrasonication and centrifugation. The supernatants were vacuum-dried, reconstituted, and analyzed using an Agilent 1290 UHPLC (Santa Clara, CA, USA) coupled with AB Triple TOF 6600 MS. Both positive and negative ion modes were employed with electrospray ionization.

Chromatographic separation was performed on an HILIC (hydrophilic interaction liquid chromatography) column using a gradient of water (with ammonium acetate/ammonia) and acetonitrile. MS (mass spectrometry) data acquisition employed IDA (Information–Dependent acquisition) mode with TOF (Time-of-Flight) MS and MS/MS scanning.

#### 2.8.2. Data Processing and Quality Control

Raw data were converted to mzML format and processed using XCMS for peak detection, alignment, and quantification. Metabolites with high missing value rates or RSD > 50% were filtered out. Missing data were imputed using KNN. Differential metabolites were identified based on fold change and statistical significance.

#### 2.8.3. Identification of Differential Metabolites (DEMs)

Differential metabolite identification was performed based on the following criteria:

Variable importance in projection (VIP) score from the OPLS-DA model ≥ 1.

*p*-value from *T*-test < 0.05.

Only metabolites meeting both criteria were considered significantly differential between groups.

#### 2.8.4. Functional Annotation and KEGG Pathway Enrichment

The annotation of metabolites was performed using an online database HMDB (human metabolome database). The KEGG pathway enrichment analysis is the same as in Section 2.7.4.

### 2.9. Transcriptomic and Metabolomic Co-Analysis

To further investigate the relationship between genes and metabolites in the muscle of largemouth bass under saline–alkaline stress, integrative multiomics analysis was performed. Two models were used for association analysis between transcriptomic and metabolomic datasets: (1) a pathway-based model, identifying KEGG pathways shared by both gene and metabolite datasets; (2) a correlation-based model, calculating Pearson correlation coefficients between gene expression levels and metabolite abundances across all groups.

### 2.10. Statistical Analysis

All data were presented as means ± standard deviation (SD). Statistical analyses were performed using SPSS version 27.0. Normality of data distribution was assessed using the Shapiro–Wilk test. One-way ANOVA followed by Tukey’s post hoc test was applied to determine significant differences among groups. A threshold of p<0.05 was considered statistically significant unless otherwise stated.

## 3. Results

### 3.1. Acute Exposure to Salinity and Alkalinity

**Figure 1:**
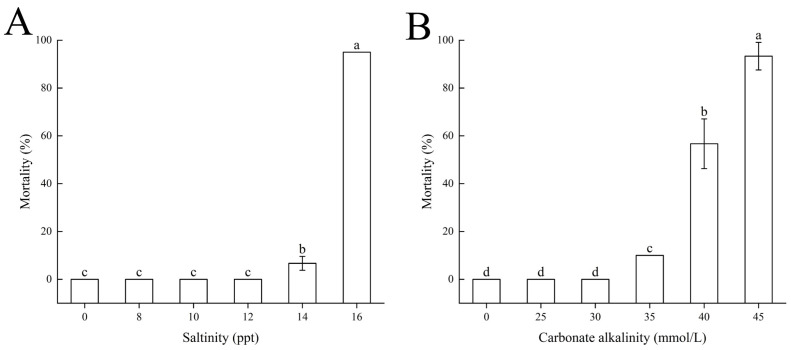
Mortality of largemouth bass under acute (**A**) salinity and (**B**) carbonate alkalinity challenges. Fish were divided into different salinity (0–16 ppt) or carbonate alkalinity (0–45 mmol/L) treatments. Mortality was assessed after a specified exposure period. Different letters indicate significant differences among treatment groups (p<0.05).

Within 96 h under salinity stress, the control group exhibited a mortality rate of 0%. When salinity increased to 14, the mortality rate of largemouth bass was 6.67 ± 2.89%, significantly higher than that of the control group (p<0.05). At a salinity of 16, the mortality rate reached 95%, significantly higher than all other groups (p<0.05). Other salinity groups had a mortality rate of 0% (Figure 1A).

The 96 h median lethal salinity concentration ($LC_{50}$) for largemouth bass was calculated using Probit regression. Based on the cumulative mortality data, the estimated $LC_{50}$ was 14.897 ppt (95% CI: 14.370–15.424 ppt), with a regression slope of 55.075 ± 7.356 and intercept of −64.388 ± 8.551. The model fit was validated by the chi-square goodness-of-fit test ($\chi^{2}$ = 2.875, df = 13, *p* = 0.998).

Under alkalinity stress, within the alkalinity range of 0–45 mmol/L, the mortality rate of largemouth bass gradually increased with rising carbonate alkalinity. The mortality rates for the control group and CA25 and CA30 treatment groups were 0%. The CA35 treatment group had a mortality rate of 10%, CA40 had 56.67 ± 10.41%, and CA45 had 93.33 ± 5.77%, significantly higher than all other groups (p<0.05) (Figure 1B).

The 96 h median lethal carbonate alkalinity concentration (${LC}_{50}$) for largemouth bass was calculated using Probit regression. The estimated $LC_{50}$ value was 39.360 mmol/L (95% CI: 38.544–40.183 mmol/L), with a regression slope of 25.714 ± 2.899 and an intercept of −41.015 ± 4.629. The model fit was validated by the chi-square goodness-of-fit test ($\chi^{2}$ = 3.987, df = 13, *p* = 0.991).

### 3.2. Long-Term Salinity and Alkalinity Stress Test

**Figure 2:**
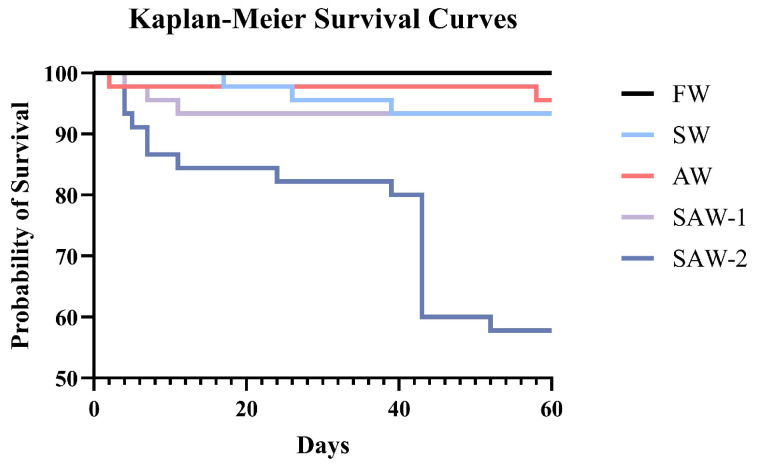
Kaplan–Meier survival curves of largemouth bass under different salinity–alkalinity treatments over a 60-day cultivation period. The FW group maintained a 100% survival rate throughout the experiment.

**Figure 3:**
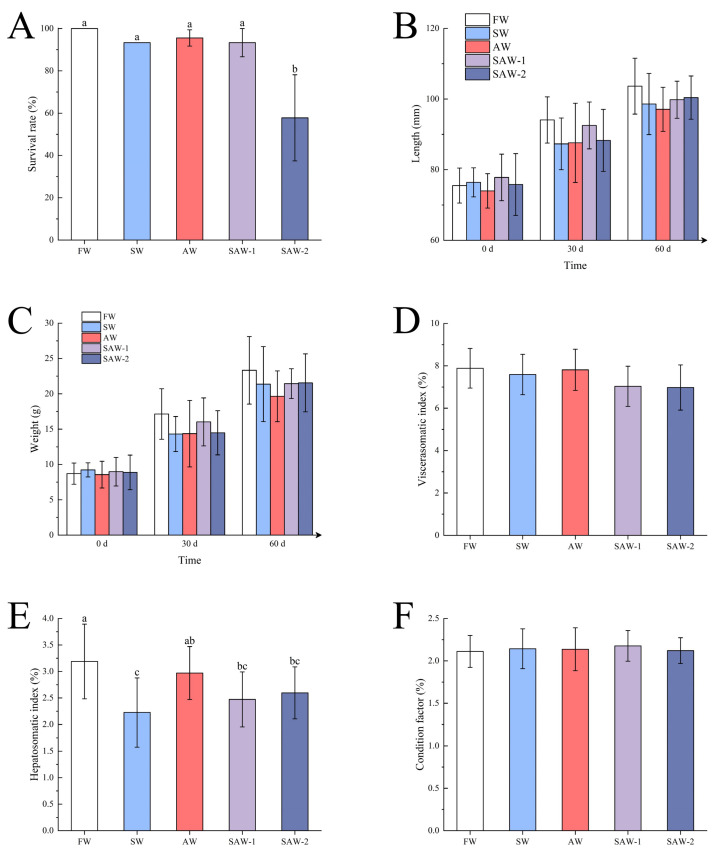
Effects of different saline–alkaline treatments on growth performance indices of largemouth bass. (**A**) Survival rate after 60 days; (**B**) body length at day 0, 30, and 60; (**C**) body weight at day 0, 30, and 60; (**D**) viscerosomatic index (VSI); (**E**) hepatosomatic index (HSI); (**F**) condition factor (CF). FW: Freshwater; SW: saline water; AW: alkaline water; SAW-1/2: two levels of saline–alkaline water. Data are presented as mean ± SD. Different letters indicate significant differences among groups (p<0.05).

Kaplan–Meier survival analysis was performed to evaluate the temporal survival patterns of largemouth bass over the 60-day cultivation period (Figure 2). The survival rate of largemouth bass in the FW group was 100%. The average survival rates in the SW, AW, and SAW-1 groups were 93.33%, 95.55 ± 3.85%, and 93.33 ± 6.67%, respectively, with no significant differences among these four groups (p>0.05) (Figure 3A). The SAW-2 group showed a marked decline in survival on the 43rd day, with a final survival rate of 57.78 ± 20.37%. Log-rank test results confirmed a significant difference in survival probability between the FW and SAW-2 groups ($\chi^{2}$ = 21.616, p<0.001).

During this period, both body length and weight of largemouth bass increased across all groups, with similar growth patterns observed. The final average body lengths were as follows: FW (103.64 ± 7.90 mm) > SAW-2 (100.40 ± 6.13 mm) > SAW-1 (99.80 ± 5.25 mm) > SW (98.58 ± 8.65 mm) > AW (97.09 ± 6.25 mm). The final average body weights were FW (23.33 ± 4.78 g) > SAW-2 (21.55 ± 4.10 g) > SAW-1 (21.44 ± 2.10 g) > SW (21.38 ± 5.30 g) > AW (19.65 ± 3.59 g). No significant differences in average body length and weight were found among the groups during each statistical period (p>0.05) (Figure 3B,C).

A linear mixed-effects model (LMM) was applied to assess the effects of treatment group (Group), time (Time), and their interaction (Group × Time) on body length and body weight of largemouth bass. Tank was included as a random factor to account for potential tank effects.

For body length, the fixed effect of Group was not significant ($F_{4,157}=1.487$, p=0.208). The effect of Time was significant ($F_{2,157}=152.376$, p<0.001). The Group × Time interaction was not significant ($F_{8,157}=0.717$, p=0.678). Estimated marginal means for Time were 7.49±0.22 cm at day 0, 10.99±0.20 cm at day 30, and 14.42±0.21 cm at day 60. Pairwise comparisons (Bonferroni-adjusted) indicated that all time points differed significantly from each other (p<0.001).

For body weight, the fixed effect of Group was not significant ($F_{4,157}=2.001$, p=0.097). The effect of Time was significant ($F_{2,157}=175.590$, p<0.001). The Group × Time interaction was not significant ($F_{8,157}=0.635$, p=0.748). Estimated marginal means for Time were 8.87±0.49 g at day 0, 15.26±0.44 g at day 30, and 21.46±0.46 g at day 60. Pairwise comparisons indicated that all time points differed significantly from each other (p<0.001).

The viscerasomatic index (VSI) values were FW (7.88 ± 0.94) > AW (7.81 ± 0.95) > SW (7.59 ± 0.97) > SAW-1 (7.03 ± 0.95) > SAW-2 (6.98 ± 1.07), without significant differences among the groups (p>0.05) (Figure 3D).

The hepatosomatic index (HSI) in the FW group (3.19 ± 0.70) was significantly higher than in the SAW-2 (2.60 ± 0.49), SAW-1 (2.47 ± 0.52), and SW (2.22 ± 0.65) groups (p<0.05), but not significantly different from the AW group (2.97 ± 0.50). No significant differences were seen among the AW, SAW-2, and SAW-1 groups (p>0.05); however, the HSI in the AW group was significantly higher than in the SW group (p<0.05), while no significant differences were found between the SAW-2, SAW-1, and SW groups (p>0.05) (Figure 3E).

The condition factor (CF) values were SAW-1 (2.18 ± 0.18%) > SW (2.142 ± 0.23%) > AW (2.137 ± 0.25%) > SAW-2 (2.12 ± 0.41%) > FW (2.11 ± 0.19%), with no significant differences observed (p>0.05) (Figure 3F).

### 3.3. Muscle Textural Properties and Sections

Muscle hardness in the SW and SAW-2 groups was significantly higher than in the FW and AW groups (p<0.05). The SAW-2 group also exhibited significantly greater muscle springiness, adhesiveness, and chewiness compared to the SW group (p<0.05); however, no significant differences were observed between the SAW-2 group and the FW, AW, and SAW-1 groups (p>0.05). No significant differences were found among all groups in terms of adhesiveness, adhesiveness extension length, cohesiveness, or chewiness (p>0.05) (Table 2).

**Figure 4:**
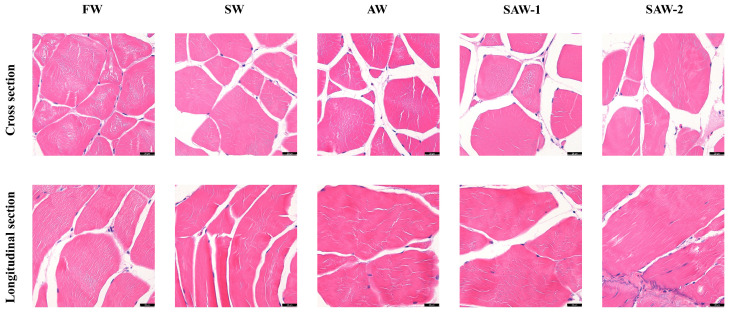
Representative hematoxylin-and-eosin (H&E)-stained sections of largemouth bass muscle tissue under different environmental treatments. Upper row: Cross-sections; lower row: longitudinal sections. FW: Freshwater; SW: saline water; AW: alkaline water; SAW-1/2: two levels of saline–alkaline water. Scale bar = 20 μm.

Due to resolution limitations of the imaging method, quantitative analysis of muscle fiber morphology (e.g., cross-sectional area, Feret diameter, or fiber density) was not performed. Instead, visual (qualitative) assessments were conducted to assess fiber arrangement and inter-fiber integrity. HE staining of skeletal muscle revealed distinct alterations in muscle fiber morphology across different treatment groups (Figure 4). In the FW group, muscle fibers exhibited well-organized polygonal or nearly circular cross-sectional profiles, with minimal inter-fiber spaces and dense perimysial connective tissue. Fiber boundaries were clearly defined, and the overall morphology indicated high structural integrity. The SW and AW groups demonstrated mild irregularities in muscle fiber shape and size, with slightly increased interstitial spaces and occasional disruption in boundary continuity. The SAW-1 group showed moderately blurred fiber boundaries, localized expansion of inter-fiber regions, and loosely packed fibers with mild disintegration of myofibrillar alignment. In the SAW-2 group, pronounced histopathological changes were observed. Muscle fibers appeared markedly irregular in shape and alignment, with substantially widened extracellular spaces and excessive accumulation of connective tissue. In the longitudinal sections, striations of sarcomeres were disrupted, and connective tissue between fibers showed signs of fragmentation and disorganization.

Notably, no morphometric quantification of fiber interstitial spaces, inter-fiber spaces or disintegration, among others, was performed, and the above findings were based on the qualitative assessment of representative histological features, which represents a limitation of the current study.

### 3.4. Analysis of Transcriptome

#### 3.4.1. Transcriptome Sequencing Overview

High-throughput transcriptome sequencing was performed on 15 cDNA libraries constructed from the muscle tissues of largemouth bass across five experimental groups. Each library generated between 35.9 and 43.9 million clean reads, resulting in a total of over 623 million reads across all samples (Table 3). The average mapping rate to the reference genome was approximately 0.06%, and the GC content ranged from 50.58% to 51.33%. These results indicate that the sequencing quality was sufficiently high to support downstream differential expression and functional enrichment analyses.

#### 3.4.2. DEG Analysis

Differentially expressed genes (DEGs) were defined as those with an adjusted *p*-value (FDR) < 0.05 and absolute $\log_{2}$ fold change ($|\log_{2}\mathrm{FC}|$) > 1. Significantly enriched GO terms were identified based on *Q* < 0.05. KEGG pathways with *Q* < 0.05 were considered significantly enriched.

**Figure 5:**
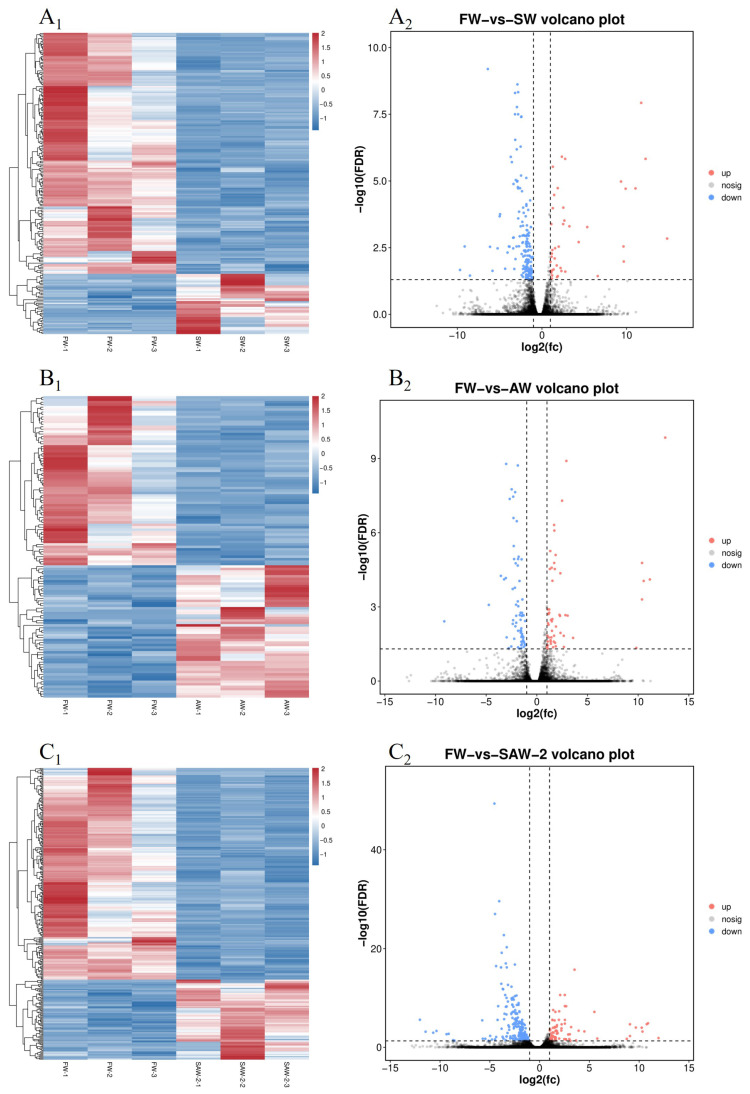
Heatmaps and volcano plots of differentially expressed genes (DEGs) in largemouth bass muscle under different treatments. ($A_{1}$–$C_{1}$) Heatmaps of DEGs for FW-vs.-SW, FW-vs.-AW, and FW-vs.-SAW-2, respectively. ($A_{2}$–$C_{2}$) Corresponding volcano plots showing upregulated (red), downregulated (blue), and non-significant (gray) genes based on $\log_{2}$(FC) and $-\log_{10}$(FDR).

**Figure 6:**
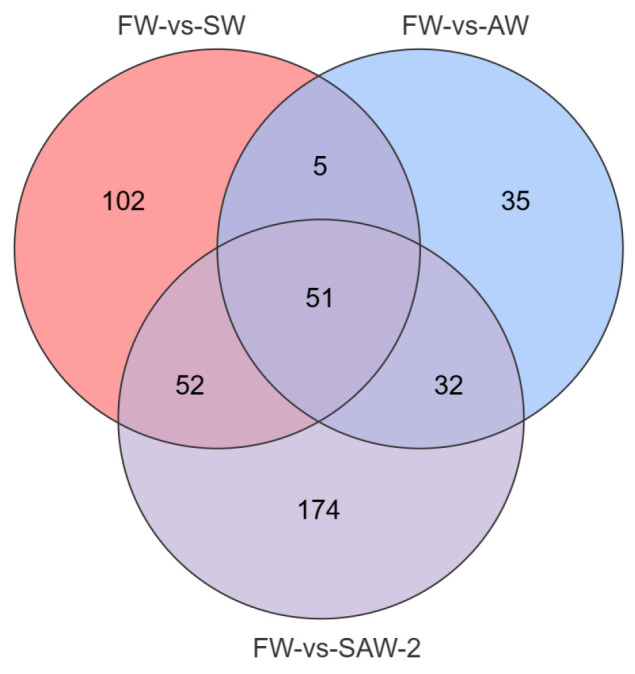
Venn diagram showing the overlap of DEGs among FW-vs.-SW, FW-vs.-AW, and FW-vs.-SAW-2 comparison groups in largemouth bass muscle tissue. Numbers indicate the count of unique or shared metabolites in each subset.

DEGs were identified among the three comparison groups, as visualized by volcano plots (Figure 5) and summarized in Table 4. The distribution of DEGs among the FW-vs.-SW, FW-vs.-AW, and FW-vs.-SAW-2 groups is illustrated in the Venn diagram (Figure 6). Specifically, 51 genes were commonly differentially expressed in all three comparisons.

**Figure 7:**
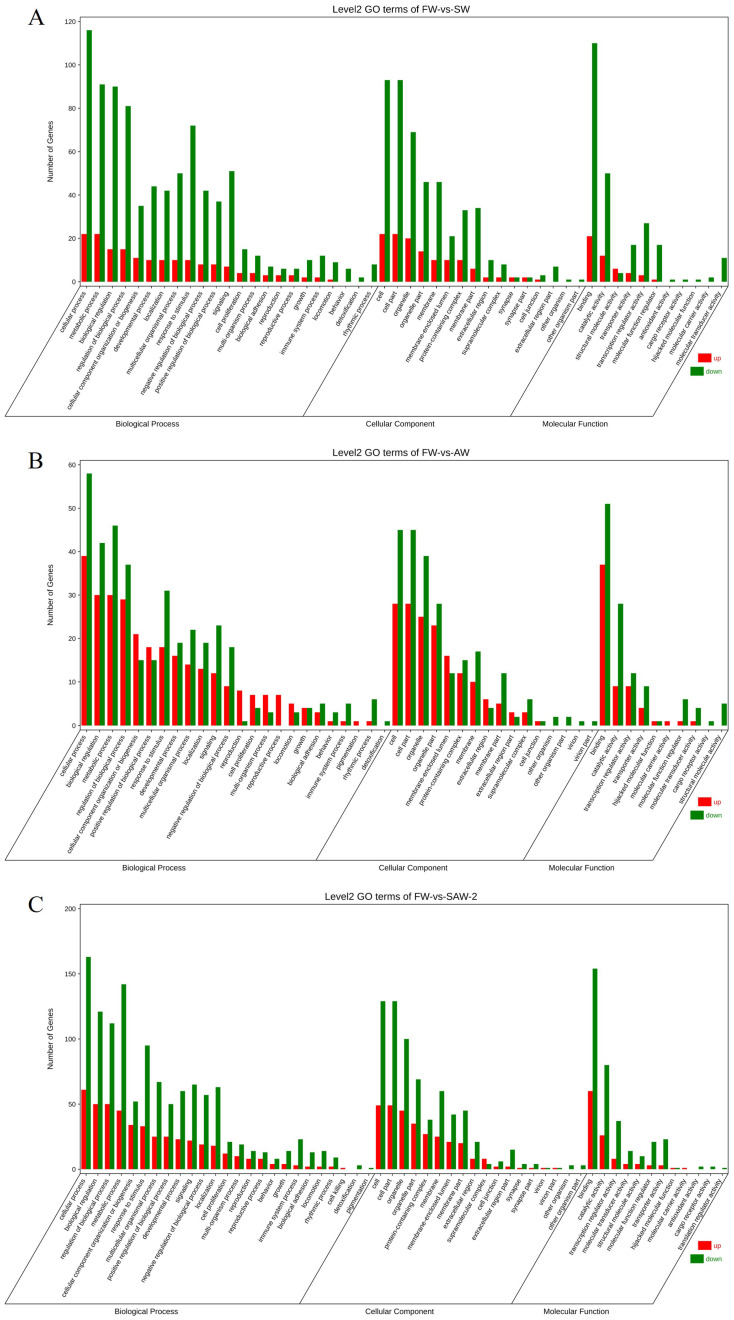
Gene Ontology (GO) level 2 classification of differentially expressed genes (DEGs) in largemouth bass muscle under different environmental conditions. (**A**) FW-vs.-SW; (**B**) FW-vs.-AW; (**C**) FW-vs.-SAW-2. The number of upregulated (red) and downregulated (green) DEGs is shown for each major GO category: biological process, cellular component, and molecular function.

**Figure 8:**
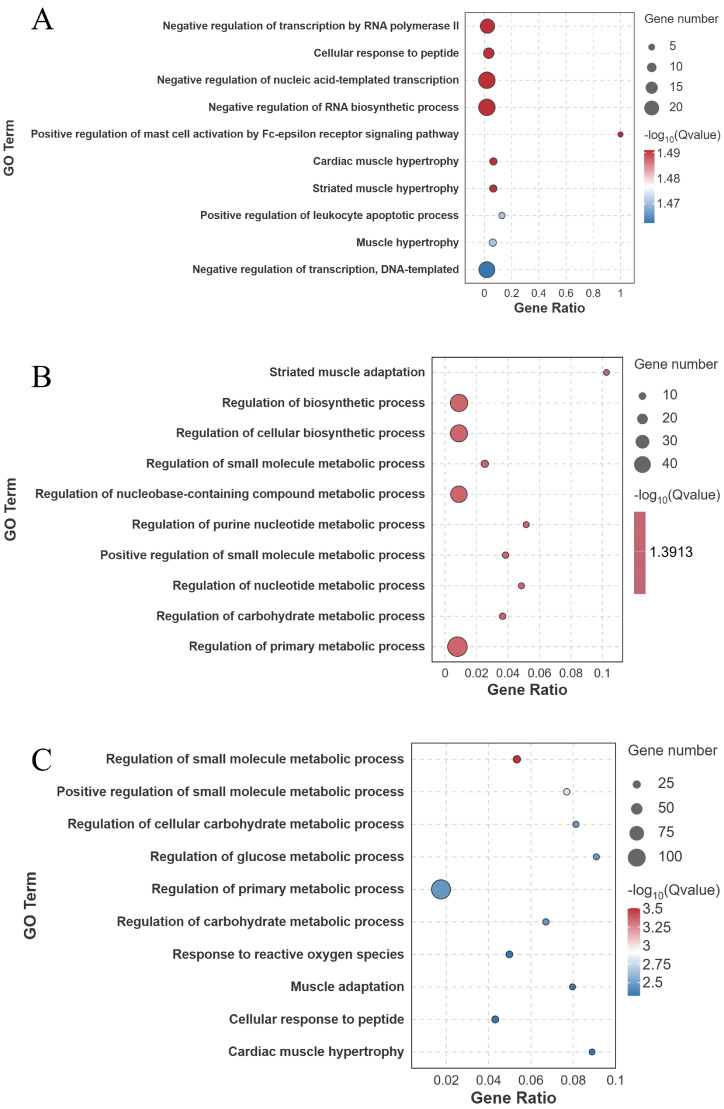
GO enrichment analysis (biological process level) of differentially expressed genes in largemouth bass muscle under different treatments. (**A**) FW-vs.-SW; (**B**) FW-vs.-AW; (**C**) FW-vs.-SAW-2. Top enriched GO terms are presented as bubble plots, with bubble size proportional to gene number and color indicating significance $-\log_{10}$(*Q*-value).

GO enrichment analysis of the DEGs revealed significant enrichment in muscle–related biological processes across all groups (Figure 7 and Figure 8).

**Figure 9:**
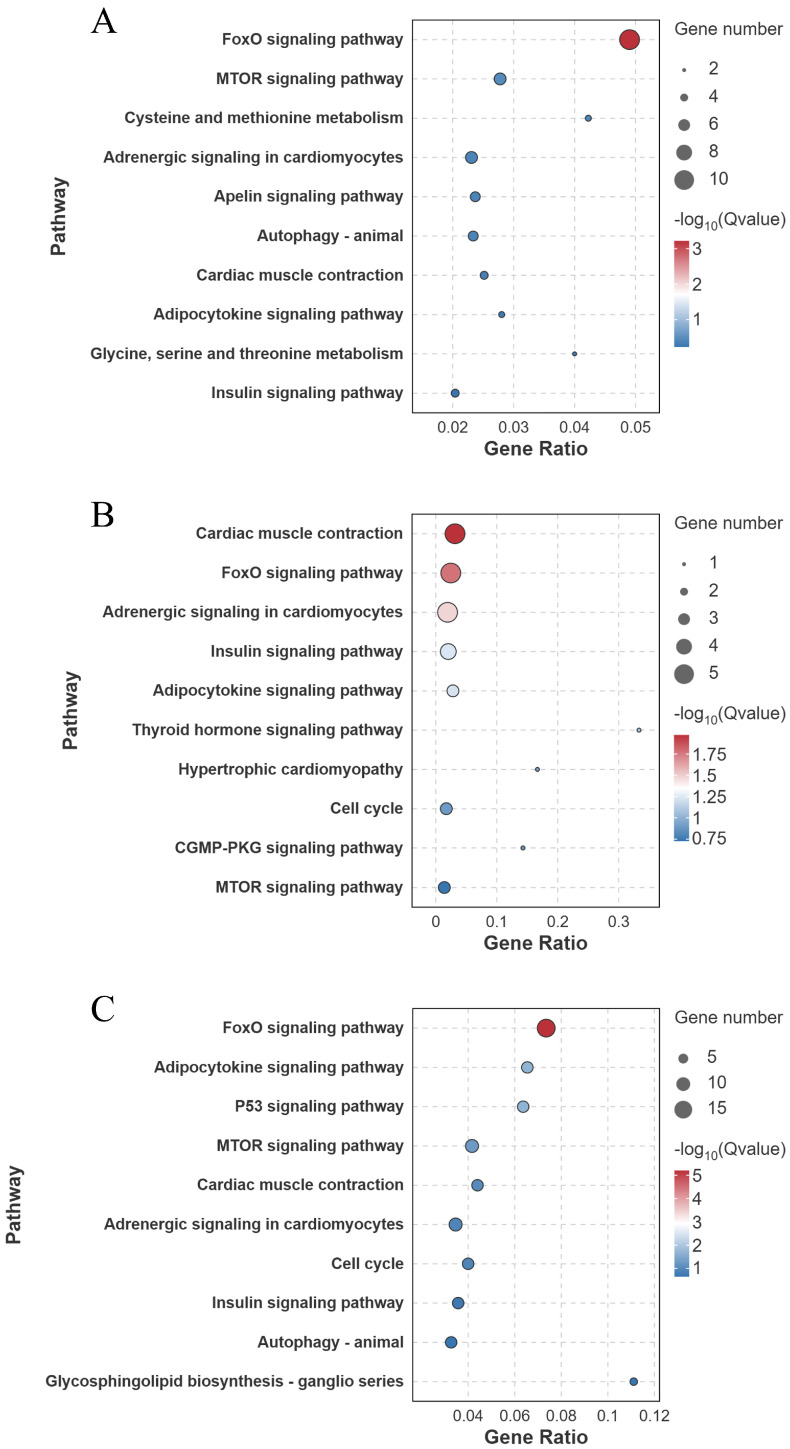
KEGG pathway enrichment analysis of differentially expressed genes (DEGs) in largemouth bass muscle under different treatments. (**A**) FW-vs.-SW; (**B**) FW-vs.-AW; (**C**) FW-vs.-SAW-2. The top enriched pathways are shown, with bubble size indicating gene count and color representing $-\log_{10}$(*Q*-value).

KEGG pathway enrichment analysis identified that in the FW-vs.-SW comparison, the FoxO signaling pathway was significantly enriched (10 genes involved) (*Q* < 0.001) (Figure 9A). In the FW-vs.-AW comparison, the cardiac muscle contraction (5 genes involved), the FoxO signaling pathway (5 genes involved), and the Adrenergic signaling in cardiomyocytes (5 genes involved) were significantly enriched (*Q* < 0.05) (Figure 9B). In the FW-vs.-SAW-2 comparison, the FoxO signaling pathway (15 genes involved), adipocytokine signaling pathway (7 genes involved), and the p53 signaling pathway (7 genes involved) were significantly enriched (*Q* < 0.05) (Figure 9C).

**Figure 10:**
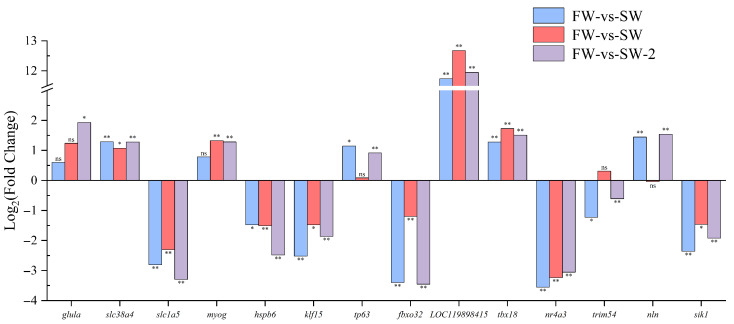
Relative expression levels $\log_{2}$(FC) of key muscle-related genes in largemouth bass under saline–alkaline stress. Each bar represents the mean $\log_{2}$(FC) for a specific comparison group—SW, AW, and SAW-2—relative to freshwater controls. Statistical significance is indicated as follows: * represents *Q* < 0.05; ** represents *Q* < 0.001; ‘ns’, not significant. Genes shown were selected based on their functional relevance to muscle development, adaptation, and stress response.

To further elucidate the molecular basis of the adaptation under saline–alkaline stress, the expression profiles of key genes were analyzed across the three experimental groups (Figure 10).

#### 3.4.3. qRT-PCR Validation

**Figure 11:**
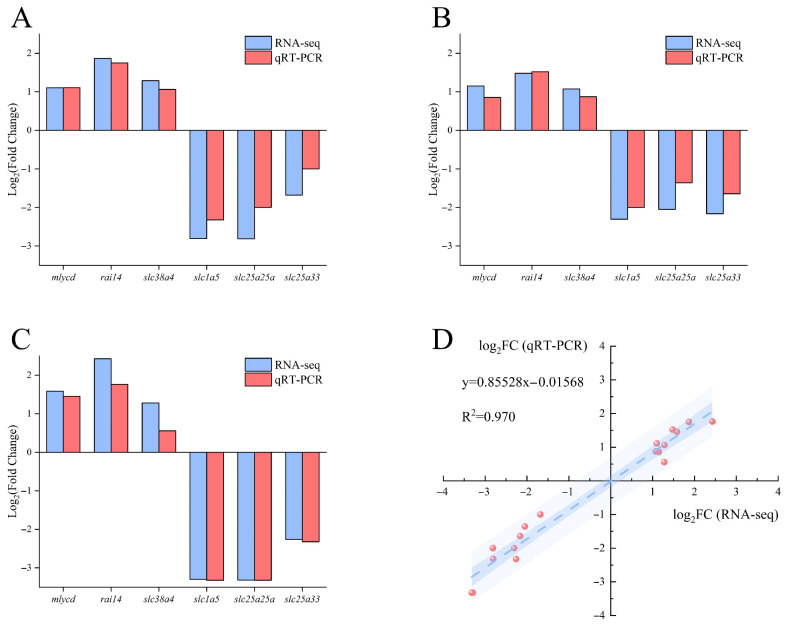
Validation of RNA-seq results by qRT-PCR for selected genes in largemouth bass muscle. (**A**) FW-vs.-SW; (**B**) FW-vs.-AW; (**C**) FW-vs.-SAW-2. Blue bars represent RNA-seq data and red bars represent qRT-PCR results for the indicated genes. Values are shown as $\log_{2}\left(\mathrm{FC}\right)$. (**D**) Correlation analysis between RNA-seq and qRT-PCR results across all comparison groups. The scatter plot shows a strong positive linear relationship between the two methods ($R^{2}=0.970$).

Comparison between qRT-PCR results and transcriptome analysis (Figure 11) revealed that the relative expression levels of the six selected differentially expressed genes showed consistent trends between RNA-seq and qRT-PCR, indicating the reliability of the transcriptomic data.

A linear regression analysis between RNA-seq and qRT-PCR results demonstrated a strong positive correlation ($R^{2}=0.970$).

### 3.5. Analysis of Metabolome

#### 3.5.1. PCA and OPLS-DA Analysis

**Figure 12:**
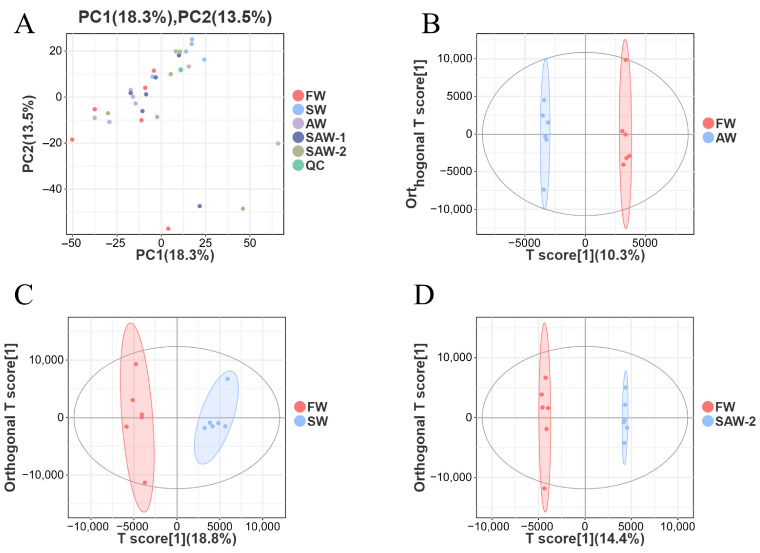
Multivariate analysis of metabolomic data from largemouth bass muscle under different treatments. (**A**) Principal component analysis (PCA) score plot showing overall sample distribution among freshwater (FW), saline water (SW), alkaline water (AW), two saline–alkaline water treatments (SAW-1, SAW-2), and quality control (QC) groups. (**B**–**D**) OPLS-DA score plots for pairwise comparisons: B: FW-vs.-SW, C: FW-vs.-AW, and D: FW-vs.-SAW-2, respectively. Each point represents a biological replicate; ellipses indicate 95% confidence intervals.

To assess the overall variation and grouping trends in the metabolomic data, principal component analysis (PCA) and orthogonal partial least squares discriminant analysis (OPLS-DA) were performed (Figure 12).

As shown in the PCA plot (Figure 12A), samples from different treatment groups were clearly separated along the first two principal components, which explained 18.3% and 13.5% of the total variance, respectively. QC samples clustered tightly together, indicating robust data quality and analytical reproducibility.

To further distinguish between the control (FW) and each treatment group, pairwise OPLS-DA models were constructed. The score plots for FW-vs.-AW, FW-vs.-SW, and FW-vs.-SAW-2 (Figure 12B–D) all exhibited clear group separation with no overlap, demonstrating that saline, alkaline, and saline–alkaline treatments each induced pronounced and distinct metabolic alterations relative to the freshwater control. The OPLS-DA model parameters for FW-vs.-AW, FW-vs.-SW, and FW-vs.-SAW-2 were $R^{2}Y$ = 0.997, 0.967, and 0.998, and $Q^{2}$ = 0.385, 0.716, and 0.811, respectively, indicating good model fit and predictive ability. The tight clustering within each group further confirms the consistency of metabolic responses among biological replicates. These results provide a solid foundation for downstream identification of differential metabolites and pathway enrichment analysis.

**Figure 13:**
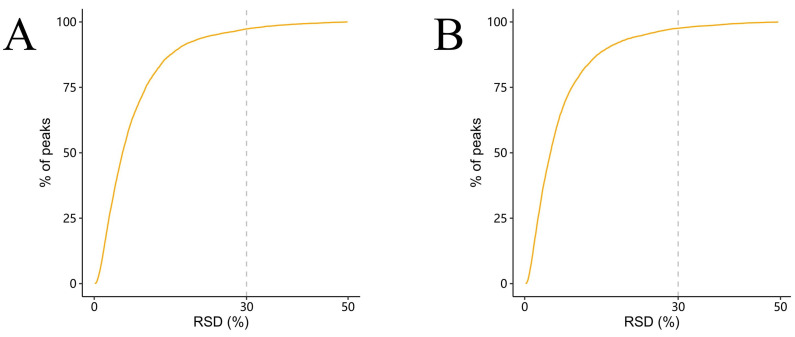
Distribution of relative standard deviations (RSDs) of peaks in QC samples. (**A**) Positive-ion mode. (**B**) Negative-ion mode. The x-axis shows the RSD (%) of individual peaks, and the y-axis represents the cumulative percentage of detected peaks.

Prior to downstream analysis, the quality and reproducibility of the metabolomic data were assessed using relative standard deviation (RSD) of quality control (QC) samples. As shown in Figure 13, more than 90% of detected peaks exhibited an RSD below 30% in both positive and negative ion modes, indicating high analytical precision and reliable metabolite quantification across the dataset.

**Figure 14:**
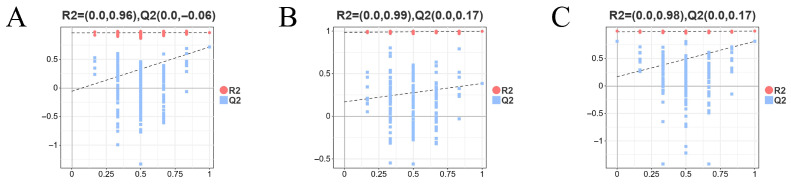
Permutation tests (200 iterations) for OPLS-DA models of FW-vs.-AW (**A**), FW-vs.-SW (**B**), and FW-vs.-SAW-2 (**C**). Red dots represent $R^{2}$ values and blue squares represent $Q^{2}$ values of permuted models.

To assess model validity and potential overfitting, permutation tests (200 iterations) were performed for each OPLS-DA model, yielding negative intercepts on the $Q^{2}$ axis and confirming model reliability (Figure 14).

#### 3.5.2. DEM Analysis

**Figure 15:**
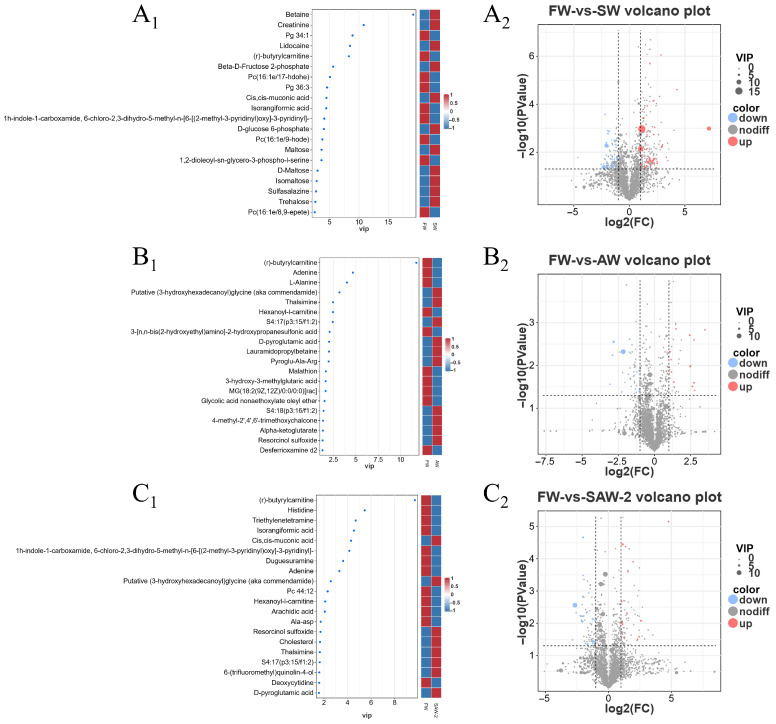
Identification of differential metabolites in muscle tissues of largemouth bass under different environmental treatments. Top 15 metabolites ranked by variable importance in projection (VIP) score from OPLS-DA for $(A_{1}$): FW-vs.-SW, ($B_{1}$): FW-vs.-AW, and ($C_{1}$): FW-vs.-SAW-2 comparisons. Heatmaps on the right represent the relative abundance of each metabolite in the compared groups. Volcano plots ($A_{2}$–$C_{2}$) showing the distribution of all detected metabolites based on $\log_{2}\left(\mathrm{FC}\right)$ and $-\log_{10}$(*p*-value) for the same comparisons.

Differential metabolites between comparison groups were selected based on the variable importance in projection (VIP) values from OPLS-DA and P-values from univariate statistical analysis (*T*-test). A total of 7411 features in positive-ion mode and 7020 features in negative-ion mode were detected. Among these, 1821 (POS) and 1504 (NEG) metabolites were annotated at MSI level 2. The selection threshold was set at VIP ≥ 1 in the OPLS-DA model and p<0.05 in the *T*-test (Figure 15) (Table 5).

The top five differential metabolites in terms of VIP values for the SW group compared to the FW group were betaine, creatinine, phosphatidylglycerol (PG34:1), lidocaine, and (r)-butyrylcarnitine (Figure 15A_1_). The top five differential metabolites for the AW group compared to FW group were (r)-butyrylcarnitine, adenine, l-alanine, putative (3-hydroxyhexadecanoyl) glycine (aka commendamide), and thalsimine Figure 15B_1_). The top five differential metabolites for the SAW-2 group compared to the FW group were (r)-butyrylcarnitine, histidine, triethylenetetramine, isorangiformic acid, and cis,cis-muconic acid (Figure 15C_1_).

**Figure 16:**
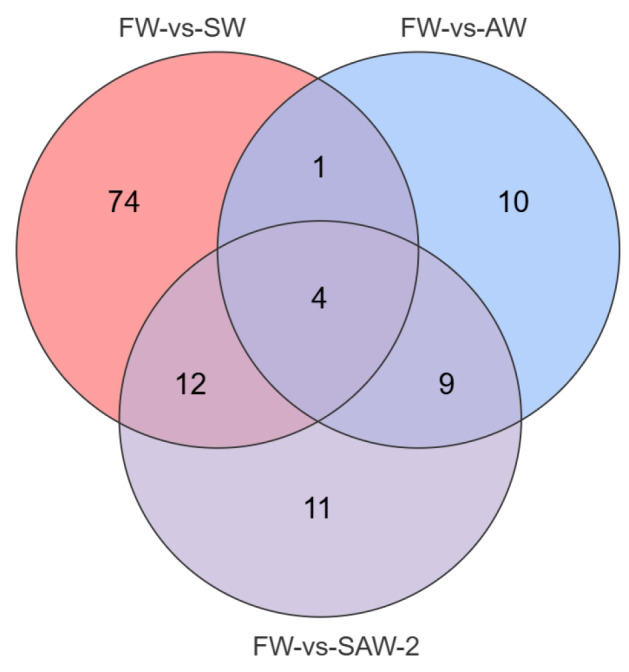
Venn diagram showing the overlap of significantly differential metabolites among FW-vs.-SW, FW-vs.-AW, and FW-vs.-SAW-2 comparison groups in largemouth bass muscle tissue. Numbers indicate the count of unique or shared metabolites in each subset.

A Venn diagram was constructed to illustrate the overlap of significantly differential metabolites among the three comparison groups (Figure 16). Among all identified differential metabolites, four were commonly altered across all three comparisons: (r)-butyrylcarnitine, hexanoyl-L-carnitine, and malathion were downregulated whereas 5-iodotubercidin was upregulated.

**Figure 17:**
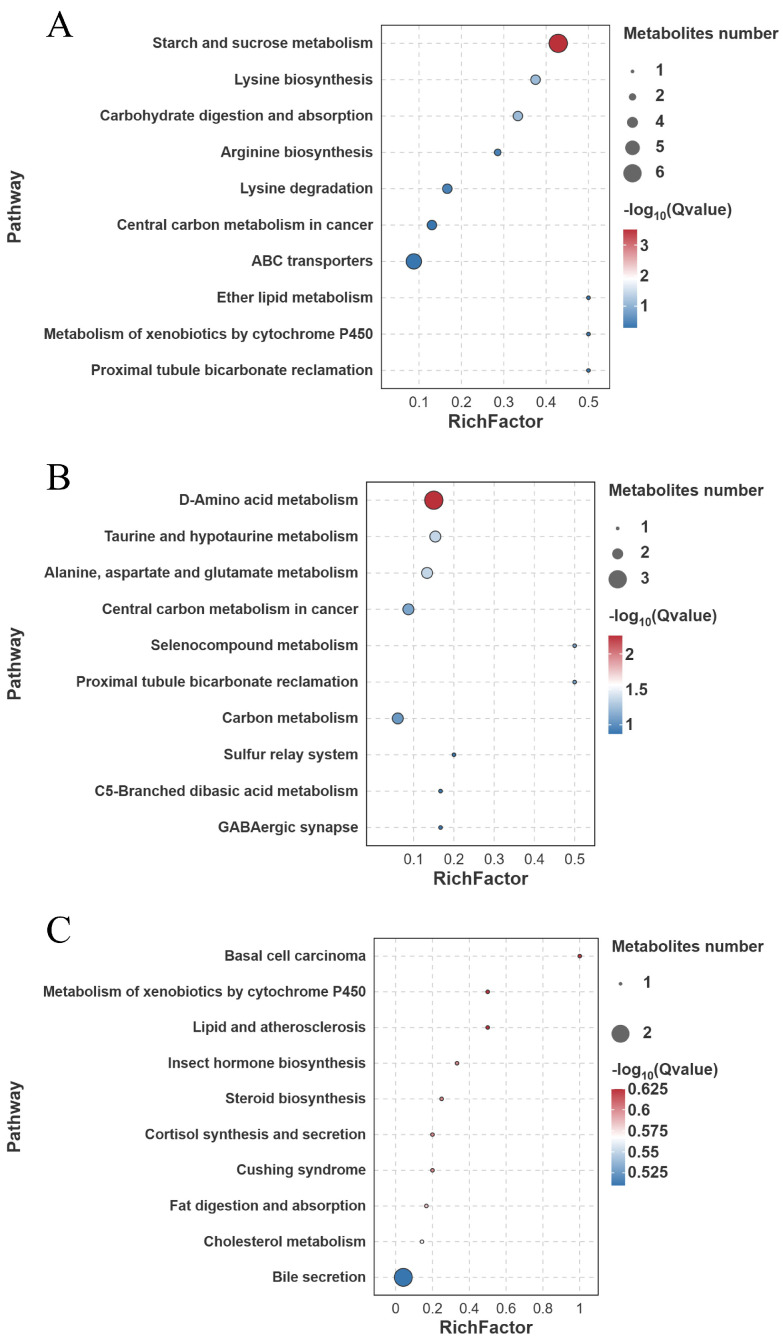
KEGG pathway enrichment analysis of differentially expressed metabolites (DEMs) in largemouth bass muscle under different treatments. (**A**) FW-vs.-SW; (**B**) FW-vs.-AW; (**C**) FW-vs.-SAW-2. The top enriched pathways are shown, with bubble size indicating gene count and color representing $-\log_{10}$(*Q*-value).

KEGG enrichment analysis of the identified differential metabolites revealed that in the FW-vs.-SW comparison, starch and sucrose metabolism was significantly enriched (6 metabolites involved) (Q<0.001) (Figure 17A). In the FW-vs.-AW comparison, D-amino acid metabolism (3 metabolites involved), taurine and hypotaurine metabolism (2 metabolites involved), and alanine, aspartate, and glutamate metabolism (2 metabolites involved) were significantly enriched (Q<0.05) (Figure 17B). In the FW-vs.-SAW-2 comparison, no pathway reached statistical significance (Q>0.05) (Figure 17C).

**Figure 18:**
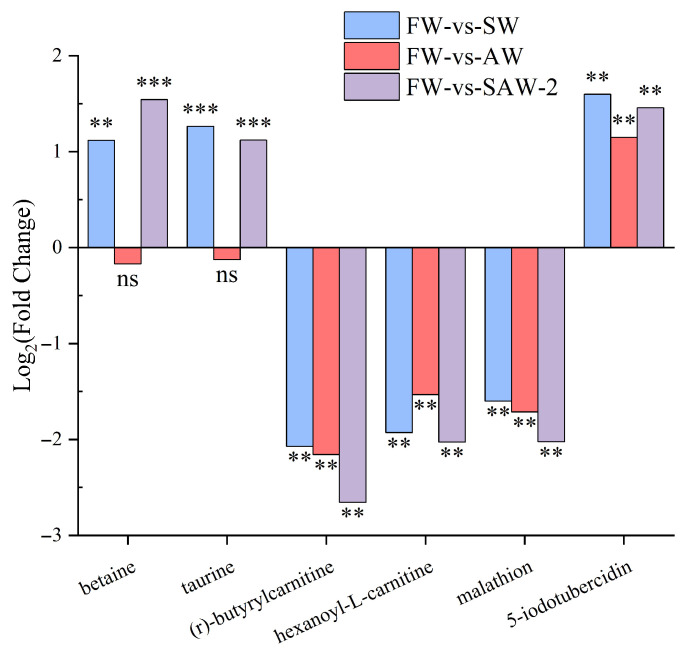
Relative expression levels $\log_{2}\left(\mathrm{FC}\right)$ of key metabolites in largemouth bass under saline–alkaline stress. Each bar represents the mean $\log_{2}\left(\mathrm{FC}\right)$ for a specific comparison group: SW, AW, and SAW-2, relative to freshwater controls. Statistical significance is indicated as follows: ** represents *Q* < 0.01; *** represents *Q* < 0.001; ‘ns’, not significant.

To further elucidate the molecular basis of adaptation under saline–alkaline stress, the expression profiles of key metabolites were analyzed across the three experimental groups (Figure 18).

### 3.6. Transcriptomic and Metabolomic Co-Analysis

Joint pathway enrichment analysis between transcriptome and metabolome data did not yield any significantly enriched pathways (Q>0.05) in all three comparison groups (FW-vs.-SW, FW-vs.-AW, and FW-vs.-SAW-2).

**Figure 19:**
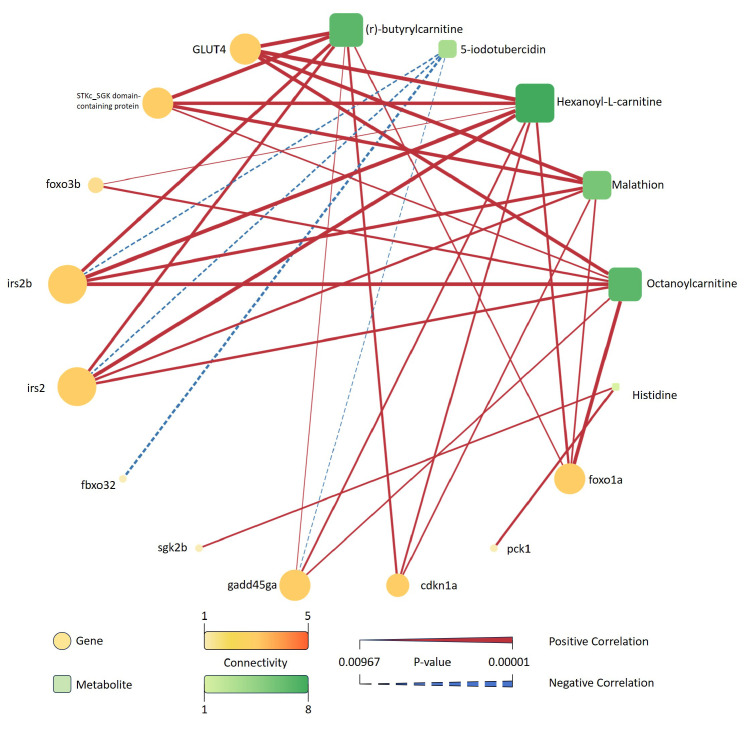
Correlation network between DEGs in the FoxO signaling pathway and selected differential metabolites under saline–alkaline stress. Genes are represented as circles and metabolites as rounded squares, with node size and color indicating their connectivity degree. Solid red lines indicate significant positive correlations, while dashed blue lines indicate significant negative correlations. The thickness of each edge corresponds to the absolute value of the correlation coefficient, and the color bar indicates the *p*-value range. Only correlations with p<0.01 are shown.

A correlation network was constructed to reveal associations between FoxO signaling pathway-related differentially expressed genes and significantly altered metabolites under saline–alkaline stress (p<0.01) (Figure 19).

In total, 11 genes and 8 metabolites were present in the network. Genes such as *irs2b*, *irs2*, and *glut4* showed the highest degree of connectivity with multiple metabolites. Among the metabolites, (r)-butyrylcarnitine, hexanoyl-L-carnitine, malathion, and octanoylcarnitine were linked to several gene nodes, indicating their central roles in the observed network structure. The majority of edges represented positive correlations, while fewer negative correlations were observed. Each gene and metabolite pair included in the network met the threshold of statistical significance (p<0.01), and the edge thickness reflected the strength of correlation.

**Figure 20:**
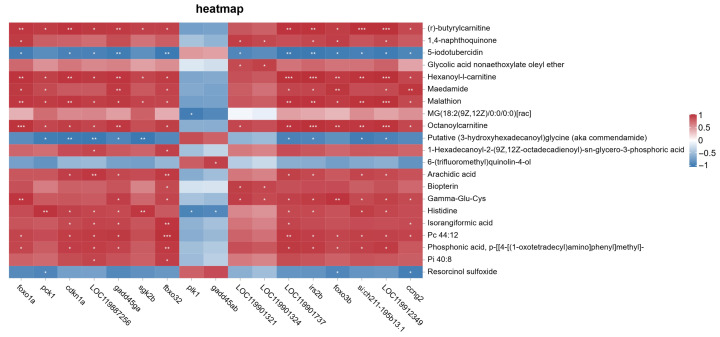
Correlation heatmap between differentially expressed genes (DEGs) involved in the FoxO signaling pathway and significantly altered metabolites in the muscle of largemouth bass under saline–alkaline stress. Pearson correlation coefficients were calculated, and values are represented by a red–blue color scale. Asterisks indicate statistical significance (* represents p<0.05, ** represents p<0.01, *** represents p<0.001). Red blocks indicate positive correlations, while blue blocks represent negative correlations.

A heatmap was generated to display the pairwise correlations between FoxO signaling pathway-related genes and significantly altered metabolites under saline–alkaline stress conditions (Figure 20).

This analysis provides a comprehensive view of the molecular associations between key genes and metabolites identified under experimental conditions. As no multiple-testing correction was applied, the results should be considered exploratory and interpreted with caution.

## 4. Discussion

### 4.1. Growth Performance, Osmoregulation, and Ammonia Metabolism

Acute toxicity assays revealed that the 96 h $LC_{50}$ values for salinity and carbonate alkalinity in largemouth bass were 14.897 ppt and 39.360 mmol/L, respectively, indicating moderate tolerance to sudden changes in these environmental factors. Under long-term exposure, the survival rate of the SAW-2 group was significantly lower than that of the other groups, demonstrating that high saline–alkaline conditions impose considerable stress on largemouth bass. Nevertheless, the surviving individuals in the SAW-2 group exhibited comparable body length and weight to those in other groups, suggesting a notable degree of adaptability.

The observed changes in survival, growth, and HSI (hepatosomatic index) reflect the physiological challenges posed by osmoregulation and ammonia excretion under saline–alkaline stress. In high-salinity and alkaline environments, fish face increased osmotic and ionic gradients, requiring enhanced ion regulation to maintain homeostasis [15]. Although no significant differential expression of canonical osmoregulatory genes (such as Na^+^/K^+^-ATPase, NKA) was detected in muscle tissue (possibly due to tissue specificity, as muscle is not a typical osmoregulatory organ), the significant reduction in HSI, especially in the SW group, suggests increased mobilization of hepatic energy reserves, likely via glycogenolysis to support the energy demands of osmoregulation [16]. Importantly, metabolomic profiling revealed significant upregulation of key osmoregulatory-related metabolites, including betaine and taurine, in the SW and SAW-2 groups. Betaine, a well-known osmoprotectant, plays a central role in mitigating damage caused by hyperosmotic stress. By forming hydrogen bonds with proteins, membrane lipids, and water molecules, betaine helps stabilize cellular structures, reduces intracellular protein denaturation, and enhances tolerance to salt stress [17]. Similarly, taurine, a non-protein amino acid, was also significantly upregulated. Taurine has been reported to regulate critical ion transport mechanisms, such as Na^+^/K^+^-ATPase and the Na^+^/Cl^−^-cotransporter (NCC), thereby supporting the maintenance of osmotic balance [18]. In addition, taurine may bolster cellular antioxidant defenses by activating enzymes such as glutathione peroxidase (GPX), superoxide dismutase (SOD), and catalase (CAT), thus further promoting stress resilience under saline–alkaline conditions [19].

**Figure 21:**
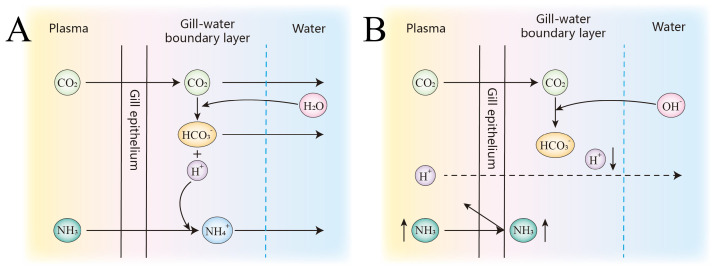
Schematic representation of ammonia metabolism and associated ${\mathrm{CO}}_{2}{\mathrm{/HCO}}_{3}^{-}$ and ${\mathrm{NH}}_{3}{\mathrm{/NH}}_{4}^{+}$ dynamics across the gill epithelium. Plasma, gill epithelium, boundary layer and external water are shown. Panel (**A**) depicts the neutral pH condition, and panel (**B**) shows the high pH/high alkalinity scenario with enhanced ${\mathrm{OH}}^{-}$ interaction. Arrows indicate diffusion and transportation pathways.

**Figure 22:**
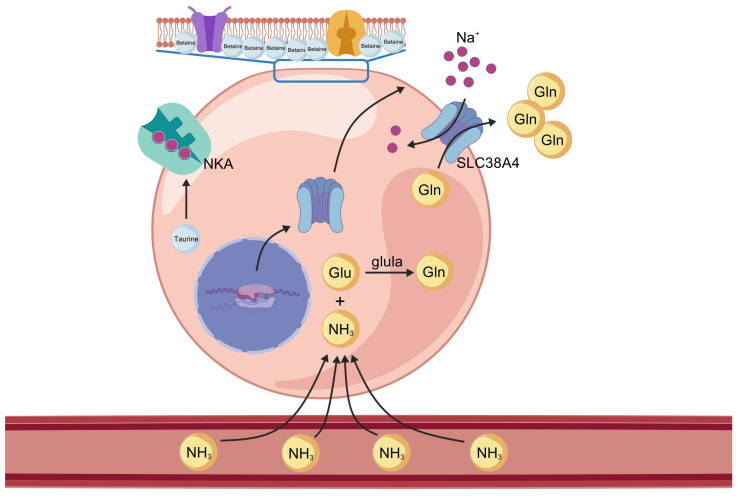
Proposed mechanism of osmoregulation and ammonia detoxification in largemouth bass muscle under saline–alkaline stress. Under high-salinity and high-alkalinity conditions, betaine accumulates as an osmoprotectant to stabilize cell structure and maintain osmotic balance. Taurine upregulates the activity of Na^+^/K^+^-ATPase (NKA), supporting ion homeostasis. Ammonia (NH_3_) enters muscle cells from the bloodstream, converting into glutamine (Gln) by glutamate–ammonia ligase a (*glula*), thereby reducing ammonia toxicity. *slc38a4* mediates the export of Gln from muscle cells into the circulation.

High alkalinity and elevated pH may disrupt normal ammonia excretion (Figure 21), leading to the accumulation of nitrogenous waste and increased metabolic burden, which could ultimately result in ammonia toxicity and mortality [3]. Fish employ multiple strategies to cope with high saline–alkaline environments, one of which is to convert accumulated endogenous ammonia into less toxic substances such as glutamine, free amino acids, or urea. In the SAW-2 group, *glula* (glutamate–ammonia ligase a, also known as glutamine synthetase) is highly significantly upregulated. This enzyme catalyzes the ATP-dependent conversion of glutamate and ammonia to glutamine, thereby preventing ammonia accumulation. This mechanism is consistent with the strategy observed in *Chalcalburnus chalcoides aralensis* when adapting to highly alkaline environments [3]. Notably, both *slc38a4* and *slc1a5* are solute carriers responsible for the transmembrane transport of glutamine [20,21]. In this study, *slc38a4* was consistently upregulated across all three comparison groups, whereas *slc1a5* was downregulated. *slc1a5* predominantly mediates the uptake of glutamine into cells, while *slc38a4* is mainly responsible for the efflux of glutamine from tissues into the circulation. The observed upregulation of *slc38a4* and downregulation of *slc1a5* under saline–alkaline stress may suggest an adaptive mechanism to enhance the export of glutamine, while restricting its unnecessary uptake, thereby contributing to nitrogen detoxification and metabolic homeostasis in largemouth bass (Figure 22).

### 4.2. Molecular Regulation of Muscle Remodeling

Based on the combined histological and texture profile analysis, the SAW-1 and SAW-2 groups exhibited varying degrees of muscle fiber irregularity, loose arrangement, widened inter-fiber spaces, and increased connective tissue in HE-stained sections. However, a significant increase in muscle hardness was observed. This was possibly due to compensatory remodeling under stress conditions, such as reduced muscle moisture content, enhanced cross-linking of myofibrillar proteins, and increased collagen synthesis, ultimately contributing to improved mechanical strength of the muscle tissue [22].

In contrast, although the SW group also showed a significant increase in hardness, its elasticity, adhesiveness, and chewiness were the lowest among all treatment groups. This may be due to osmotic stress induced by high salinity, which can cause protein denaturation or degradation, thereby compromising the integrity of structural proteins and the elastic network within muscle tissue [23].

To further dissect the molecular underpinnings of these structural and mechanical changes, transcriptomic analysis was conducted. GO enrichment reveals that saline–alkaline stress induces substantial changes in the expression of muscle-related genes in largemouth bass, as reflected by the enrichment of biological processes such as muscle hypertrophy, regulation of muscle hypertrophy, and muscle adaptation. *Myog* (myogenin), a key transcription factor in muscle cell differentiation, was upregulated, suggesting that the fish initiate compensatory myogenesis to maintain or restore muscle structure in response to environmental challenge. This upregulation may reflect an adaptive strategy to offset potential muscle loss or damage caused by osmotic stress [24]. Downregulation of *hspb6*, a small heat shock protein, further suggests that muscle cells may reduce reliance on certain protective pathways during prolonged exposure to adverse conditions, reallocating resources to more critical cellular processes [25].

A distinct anti-atrophy program also appeared to be activated. For example, *klf15* was downregulated, implying suppression of pathways involved in muscle metabolism and catabolism [26], while *tp63* was upregulated, potentially enhancing muscle cell proliferation or differentiation under stress. One of the most striking observations is the significant downregulation of *fbxo32* (atrogin-1), a key E3 ubiquitin ligase involved in muscle protein degradation and a downstream effector in the FoxO signaling pathway. Since atrogin-1 is closely linked to muscle atrophy [25], its reduced expression may represent an adaptive attempt by the organism to limit unnecessary muscle breakdown under saline–alkaline stress. This is consistent with the enrichment of the FoxO signaling pathway seen in KEGG analysis, further supporting a model in which the FoxO-atrogin-1 axis is central to muscle maintenance in harsh environments.

Within this broader transcriptional landscape, the co-upregulation of *loc119898415* and *tbx18* may represent a central regulatory axis coordinating chromatin remodeling, lineage commitment, and muscle fiber regeneration under saline–alkaline stress. *loc119898415* encodes a putative component of the SIN3 histone deacetylase (HDAC) complex, which is known to repress transcription through chromatin condensation and epigenetic silencing [27]. This mechanism is essential for stabilizing muscle progenitor identity by inhibiting non-myogenic programs and maintaining a poised but undifferentiated state. Under stress conditions, such epigenetic tightening may serve as a protective checkpoint, preventing aberrant fate decisions while priming cells for controlled regeneration [28]. Downstream of this chromatin-level control, *tbx18*, a transcription factor that guides mesenchymal-to-myogenic transition, may act as a molecular switch to direct lineage execution [29]. Its upregulation suggests the initiation of progenitor differentiation toward muscle-specific fates. Supporting this cascade, *tp63*, a known promoter of cell proliferation and differentiation, was also upregulated, potentially facilitating the expansion of regenerative muscle cell pools [30]. Together, these genes outline a coherent developmental program: from epigenetic gating (*loc119898415*), through lineage instruction (*tbx18*), to regenerative execution (*tp63*, *Myog*).

Other genes, such as *nr4a3* and *trim54*, were also downregulated, indicating broader remodeling of regulatory networks related to muscle plasticity and protein turnover [31], while the upregulation of *nln* (neurolysin) suggests alterations in peptide processing that may contribute to muscle adaptation or stress recovery [32]. *Sik1* was downregulated in all relevant comparisons, pointing to reduced activity in salt-inducible kinase signaling and possibly indicating shifts in muscle cell energy metabolism or ionic regulation [33].

### 4.3. Regulation of FoxO Signaling Pathway in Muscle Osmoadaptation

**Figure 23:**
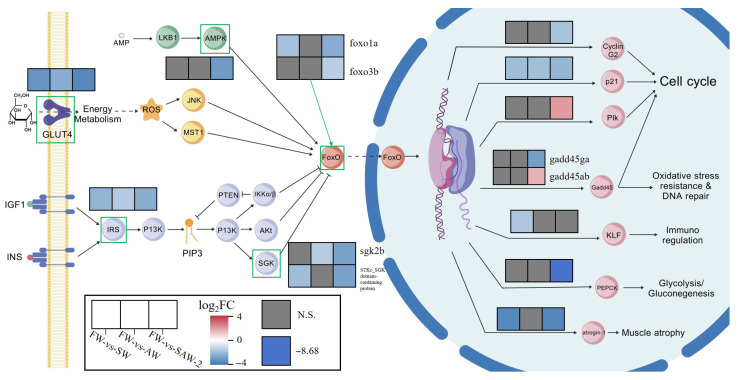
Integrated visualization of gene expression changes in the FoxO signaling pathway in the muscle tissue of largemouth bass under different salinity–alkalinity stress conditions. For each gene, three adjacent color blocks represent the $log_{2}\left(FC\right)$ in the FW-vs.-SW, FW-vs.-AW, and FW-vs.-SAW-2 groups, respectively. Color gradients indicate the direction and magnitude of gene expression changes. Only genes with significant expression changes (*p* < 0.05) are shown in color, while gray blocks denote genes with no significant differential expression (*p* > 0.05).

In KEGG enrichment analysis, the FoxO signaling pathway is significantly enriched in the SW, AW, and SAW-2 groups (Figure 23). The FoxO signaling pathway is a highly conserved intracellular signaling cascade that regulates diverse cellular processes, including cell survival, oxidative stress response, metabolism, and apoptosis [34]. In teleosts, FoxO transcription factors have been shown to play pivotal roles in mediating environmental stress adaptation, particularly under osmotic and ionic challenges [35].

At the upstream level, *glut4* (glucose transporter type 4), a target of insulin signaling and a key modulator of glucose uptake [36], was found to be downregulated. GLUT4 facilitates passive glucose diffusion, yet its membrane translocation is tightly regulated by insulin signaling and cytoskeletal dynamics. This reduction in *glut4* expression may indicate impaired insulin sensitivity and glucose transport, which are common adaptive responses in fish muscle under hyperosmotic conditions [37]. Such downregulation likely represents a metabolic reallocation strategy. By decreasing GLUT4-mediated glucose entry, skeletal muscle restricts glucose utilization, possibly to prioritize glucose supply to vital organs under prolonged salinity–alkalinity stress. The muscle may also reduce osmotic load and avoid excessive intracellular accumulation of ions and metabolites, thereby contributing to cellular homeostasis during prolonged salinity–alkalinity stress [38]. However, this interpretation is based on transcriptional data only. Functional insulin sensitivity was not directly assessed in this study. Future work should evaluate GLUT4 translocation to the plasma membrane and the phosphorylation status of Akt (protein kinase B) to confirm changes in insulin signaling.

Furthermore, components of the insulin signaling axis (IRS, PI3K-Akt pathway) also exhibited suppressed expression, indicating that the crosstalk between IRS/PI3K/Akt and FoxO signaling may be compromised during stress [39]. This axis normally promotes the phosphorylation and cytoplasmic retention of FoxO transcription factors, thereby inhibiting their activity [34]. The downregulation observed here is likely to increase the nuclear localization of FoxO proteins, but interestingly, the FoxO genes themselves (*foxo1a*, *foxo3b*) were downregulated, implying a complex regulatory feedback possibly aiming to limit excessive activation of catabolic or pro-apoptotic genes in muscle cells during chronic stress [25].

At the level of downstream effectors, genes such as *gadd45*, *plk*, and others involved in cell cycle arrest, DNA repair, and apoptosis also displayed reduced expression. This pattern suggests that muscle tissue under salinity–alkalinity stress may suppress unnecessary cell turnover and DNA repair activities to conserve resources, while avoiding excessive apoptosis that could lead to tissue degeneration [40].

Such a regulatory mode may help maintain muscle structure and function by prioritizing cellular stability and energy conservation over growth and proliferation during prolonged environmental stress and withstand fluctuating or challenging osmotic environments. Together, these findings support a stress adaptation model in which largemouth bass muscle undergoes a shift from anabolic growth toward cellular maintenance and energy conservation, suggesting that this transition may be orchestrated by a selective modulation of the FoxO signaling network.

### 4.4. Energy Metabolism and Membrane Remodeling

In all the DEMs, only four metabolites—(r)-butyrylcarnitine, 5-iodotubercidin, hexanoyl-l-carnitine, and malathion—were consistently and significantly altered across all experimental comparisons (FW-vs.-SW, FW-vs.-AW, and FW-vs.-SAW-2). These findings suggest that these metabolites may serve as universal metabolic markers or mediators of largemouth bass adaptation to saline–alkaline stress.

Both (r)-butyrylcarnitine and hexanoyl-l-carnitine are short- and medium-chain acylcarnitine derivatives (C4 and C6, respectively) involved in the transport of fatty acids into mitochondria for β-oxidation. Their consistent reduction across all stress comparisons indicates a broad suppression of mitochondrial lipid catabolism, affecting multiple chain lengths. This downregulation of acylcarnitines likely reflects inhibition of fatty acid β-oxidation, a key adaptive response under osmotic or alkaline stress. This metabolic adjustment may reflect an energy conservation strategy, as excessive β-oxidation could increase the risk of oxidative stress or generate metabolic intermediates that are not efficiently processed under osmotic or alkaline stress [41]. Suppression of acylcarnitine turnover might also help stabilize cellular homeostasis by preventing the accumulation of potentially toxic intermediates or reducing the demand for mitochondrial ATP production when resources are limited [42]. Such shifts are commonly observed in fish exposed to environmental stressors, where metabolic flexibility is crucial for survival [43].

5-Iodotubercidin, a nucleoside analog, was upregulated in all comparisons. This compound is known to act as an inhibitor of adenosine kinase and may interfere with purine metabolism and nucleotide signaling [44]. Its accumulation under stress conditions could indicate an adaptive remodeling of nucleotide metabolism, possibly to regulate energy balance, modulate signal transduction pathways, or provide cytoprotective effects. Elevated levels of nucleoside analogs have been linked to DNA repair, cellular stress signaling, and adaptive metabolic responses in various organisms [45]. The upregulation of 5-iodotubercidin might therefore represent an integral component of the largemouth bass’s strategy to maintain genomic and metabolic integrity during sustained environmental challenge.

Malathion, an organophosphate compound, exhibited significant downregulation in all stress groups. It is most likely an exogenous environmental contaminant rather than an endogenous metabolite. It and its derivatives can be present as environmental contaminants or as products of endogenous detoxification pathways. The decrease in malathion levels may reflect enhanced detoxification and excretion mechanisms activated under stress [46], or a shift in metabolic priorities away from xenobiotic processing toward essential physiological functions. This response may help limit the metabolic burden of toxicant handling when energy and cellular resources are redirected to coping with osmotic and alkaline stress. The observed reduction also suggests a general enhancement of metabolic resilience and environmental adaptability in largemouth bass.

The metabolomic analysis revealed pronounced changes in a variety of phospholipid species, including phosphatidylcholine and glycerophospholipids, and others, across different stress comparisons (Figure 15). These alterations in phospholipid abundance suggest active membrane remodeling processes in largemouth bass muscle in response to saline–alkaline stress [47]. Phospholipids are fundamental structural components of biological membranes and play a critical role in maintaining membrane fluidity, integrity, and function [48]. Under osmotic and ionic stress, adaptive remodeling of membrane lipids can serve multiple purposes: (1) stabilizing membrane structure against changes in external tonicity; (2) modulating membrane permeability to ions and water; and (3) providing substrates for signaling lipids involved in stress responses. The observed up– and down–regulation of distinct phospholipid molecules may reflect selective adjustments aimed at optimizing the biophysical properties of muscle cell membranes under stress. Such remodeling may also impact the function of membrane-bound proteins, including ion transporters, receptors, and enzymes critical for muscle homeostasis. For instance, changes in phosphatidylglycerol and other glycerophospholipids could influence mitochondrial membrane dynamics, thereby affecting cellular energy metabolism [49]. Given the centrality of membrane composition for mitochondrial and cellular function, these lipid changes may interact with the transcriptional regulation of metabolic genes. The differential regulation of phospholipids observed in this study supports the hypothesis that membrane adaptation is a key component of largemouth bass muscle resilience to saline–alkaline environments. This interpretation is consistent with previous lipidomics studies in teleosts, which have shown that changes in phospholipid profiles contribute to osmoregulation and stress tolerance under challenging aquatic conditions [50,51].

The integrative network analysis between FoxO signaling pathway-related genes and significantly altered metabolites (Figure 19) revealed several high-connectivity nodes and key molecular hubs. Among the metabolites, acylcarnitines such as (r)-butyrylcarnitine, hexanoyl-L-carnitine, and octanoylcarnitine (C8) demonstrated extensive correlations with multiple genes, including *glut4*, *irs2*, and *gadd45ga*. These metabolites serve as sensitive indicators of fatty acid import into mitochondria and β-oxidation flux, linking energy metabolism directly to transcriptional regulation [52]. Notably, *glut4* and *irs2*, central components of the insulin signaling pathway, exhibited the highest degree of connectivity, suggesting that modulation of glucose uptake and insulin sensitivity is tightly integrated with fatty acid metabolism under stress [53]. The predominance of positive correlations in the network indicates a coordinated up- or downregulation of gene expression and metabolite abundance, reinforcing the notion of a system-level adaptive response. The network also highlights the crosstalk between energy metabolism and cell cycle/oxidative stress pathways, as evidenced by gene nodes such as *gadd45ga* and *cdkn1a*, which connect to both acylcarnitines and phospholipid metabolites. This structure underscores the multifaceted nature of the stress response, where metabolic reprogramming, membrane adaptation, and transcriptional control are interdependent [54].

## 5. Conclusions

This study provides a comprehensive multi-level analysis of largemouth bass adaptation to saline, alkaline, and combined saline–alkaline stress, integrating physiological, histological, transcriptomic, and metabolomic perspectives. The results reveal that although overall growth performance was maintained, survival and muscle structure were compromised under the highest combined stress (SAW-2). Saline–alkaline challenges triggered adaptive changes at multiple levels: muscle hardness increased, fiber arrangement became irregular, and energy reserves in the liver could mobilize.

The FoxO signaling pathway serves as a central regulatory hub, orchestrating muscle maintenance, metabolic reprogramming, and cellular stress responses. Integrative omics analysis highlighted consistent changes in both gene expression and metabolite profiles, particularly involving insulin signaling, nitrogen metabolism, and fatty acid oxidation. Genes critical for nitrogen detoxification, *glula* and *slc38a4*, were significantly upregulated, highlighting an enhanced capacity for ammonia assimilation and transport as an essential adaptation mechanism under stress. Metabolomic analysis further revealed that accumulation of osmoprotectants (betaine and taurine), membrane lipid remodeling, and the suppression of fatty acid β-oxidation are key strategies for coping with environmental stress. Furthermore, integrative transcriptomic and metabolomic network analysis identified *glut4* and *irs2* as central nodes linking insulin signaling with shifts in fatty acid metabolism, as reflected by the coordinated changes in acylcarnitines and nucleotide metabolism.

From an applied perspective, these findings provide a molecular basis for selective breeding programs targeting stress-resilient traits in largemouth bass, such as improved ammonia detoxification and metabolic flexibility. Moreover, managing dietary inputs or environmental buffering strategies to support FoxO and insulin signaling could enhance fish health in saline–alkaline aquaculture systems.

Future research should include functional validation of key pathways identified here, such as phospho-Akt quantification, GLUT4 translocation assays, and targeted gene knockdowns to confirm causal roles.

In summary, this multiomics approach revealed a tightly coordinated adaptive strategy encompassing energy reallocation, membrane and muscle remodeling, nitrogen detoxification and osmotic protection, which together underpin the physiological resilience of largemouth bass in saline–alkaline environments. These insights not only deepen our understanding of teleost adaptation under compound stress but also offer practical guidance for sustainable aquaculture.

## Figures and Tables

**Table 1:** Sequences of primers used for quantitative real-time PCR (qRT-PCR) analysis. Primer names, sequences (5’-3’), and expected amplicon sizes (bp) are listed for each target gene.

Gene	Gene ID	Primer	Primer Sequence (5’-3’)	Size (bp)
*slc1a5*	ncbi_119883450	*slc1a5*-F	GCGGGTGAAGAGGATAGTGC	233
		*slc1a5*-R	CCAGGGGCTTTAGGGTCAAT	
*slc38a4*	ncbi_119918555	*slc38a4*-F	CTGGACACGCCACTTCGCT	202
		*slc38a4*-R	GTCGTGCCCGAATTTTCTGA	
*slc25a25a*	ncbi_119892260	*slc25a25a*-F	TCAGCCTTTAGCTGCTTTACG	165
		*slc25a25a*-R	GGTGTTTTCGTGCCCATC	
*slc25a33*	ncbi_119905602	*slc25a33*-F	GGGTTGTAGTGAGGATGGG	95
		*slc25a33*-R	AGACTGTTCTGGTGAGTGG	
*mlycd*	ncbi_119894455	*mlycd*-F	TGCTACTGGGGCTTTTGCGT	184
		*mlycd*-R	TCTTGTTGCTGATGGGTTGAA	
*rai14*	ncbi_119914322	*rai14*-F	ACAGGTTCGCCGGTGAACAA	211
		*rai14*-R	GCTTCTGACTGGGCTTTCCT	
*gapdh*	ncbi_119897949	*gapdh*-F	GCAGAAACCCGGCAAATA	236
		*gapdh*-R	TCAGGTCCAGACACACGGT	

**Table 2:** Muscle textural properties of largemouth bass under different treatments. Hardness, adhesiveness, stringiness, cohesiveness, springiness, gumminess, and chewiness were measured. Data are shown as mean ± SD (*n* = 6 fish per group). Measurements were conducted at the individual fish level (two fish per tank, three replicate tanks). Different letters indicate statistically significant differences among groups (p<0.05).

Group	Hardness (N)	Adhesiveness (N·m)	Stringiness Length (mm)	Cohesiveness	Springiness (mm)	Gumminess (N)	Chewiness (mJ)
FW	12.83 ± ${2.48\;}^{b}$	0.07 ± 0.04	0.97 ± 0.00	0.25 ± 0.06	0.42 ± ${0.18\;}^{ab}$	3.38 ± ${0.72\;}^{ab}$	1.45 ± 1.11
SW	18.58 ± ${1.50\;}^{a}$	0.05 ± 0.01	0.95 ± 0.01	0.25 ± 0.06	0.25 ± ${0.04\;}^{b}$	2.45 ± ${0.90\;}^{b}$	1.21 ± 0.49
AW	13.55 ± ${2.69\;}^{b}$	0.08 ± 0.03	0.93 ± 0.01	0.28 ± 0.05	0.37 ± ${0.08\;}^{ab}$	4.03 ± ${1.39\;}^{ab}$	1.35 ± 0.55
SAW-1	16.08 ± ${4.17\;}^{ab}$	0.08 ± 0.02	0.95 ± 0.03	0.23 ± 0.05	0.36 ± ${0.08\;}^{ab}$	3.68 ± ${1.35\;}^{ab}$	1.48 ± 0.83
SAW-2	19.85 ± ${5.21\;}^{a}$	0.08 ± 0.02	0.95 ± 0.01	0.28 ± 0.05	0.43 ± ${0.03\;}^{a}$	5.20 ± ${1.17\;}^{a}$	2.30 ± 0.77

**Table 3:** Summary of transcriptome sequencing quality for each sample, including total number of clean reads, mapping rate to the reference genome, and GC content.

Sample	Clean Reads	Mapped Reads (%)	GC Content (%)
FW-1	39,904,406	0.05	50.62
FW-2	35,962,314	0.05	50.71
FW-3	38,064,170	0.05	51.13
SW-1	43,923,728	0.09	51.14
SW-2	39,627,764	0.05	50.69
SW-3	42,404,904	0.09	51.19
AW-1	43,857,806	0.08	50.73
AW-2	37,661,316	0.05	50.87
AW-3	43,386,644	0.04	50.58
SAW-1-1	40,304,858	0.06	50.85
SAW-1-2	42,623,044	0.06	50.94
SAW-1-3	40,122,084	0.05	50.92
SAW-2-1	41,362,832	0.07	51.08
SAW-2-2	40,841,490	0.05	50.92
SAW-2-3	42,512,590	0.07	51.33

**Table 4:** Summary of differentially expressed genes (DEGs) and Gene Ontology (GO) enrichment analysis in largemouth bass muscle under different treatments. The total number of DEGs (upregulated and downregulated) and the number of significantly enriched GO terms for biological process, cellular component, and molecular function categories are presented for comparison.

Group	DEGs			GO Enrichment			
	Total	Up	Down	Total	Biology Process	Cell Component	Molecular Function
FW-vs.-SW	210	42	168	671	552	27	92
FW-vs.-AW	123	54	69	505	373	54	78
FW-vs.-SAW-2	309	85	224	855	677	44	134

**Table 5:** Summary of differentially expressed metabolites (DEMs) identified in muscle tissue of largemouth bass under different environmental treatments. The number of upregulated and downregulated DEMs is presented for each comparison group.

Group	Total	Up	Down
FW-vs.-SW	91	42	49
FW-vs.-AW	24	13	11
FW-vs.-SAW-2	36	13	23

## Data Availability

The datasets will be made available on request.
